# Review of Wearable Sensor-Based Health Monitoring Glove Devices for Rheumatoid Arthritis

**DOI:** 10.3390/s21051576

**Published:** 2021-02-24

**Authors:** Jeffrey Henderson, Joan Condell, James Connolly, Daniel Kelly, Kevin Curran

**Affiliations:** 1 School of Computing, Engineering and Intelligent System, Ulster University Magee Campus, Northland Rd, BT48 7JL Londonderry, Ireland; j.condell@ulster.ac.uk (J.C.); d.kelly@ulster.ac.uk (D.K.); kj.curran@ulster.ac.uk (K.C.); 2 School of Science, Letterkenny Institute of Technology, Port Rd, Gortlee, F92 FC93 Letterkenny, Ireland; james.connolly@lyit.ie

**Keywords:** rheumatoid arthritis, smart sensing, data gloves, joint measurement, rehabilitation, range of motion

## Abstract

Early detection of Rheumatoid Arthritis (RA) and other neurological conditions is vital for effective treatment. Existing methods of detecting RA rely on observation, questionnaires, and physical measurement, each with their own weaknesses. Pharmaceutical medications and procedures aim to reduce the debilitating effect, preventing the progression of the illness and bringing the condition into remission. There is still a great deal of ambiguity around patient diagnosis, as the difficulty of measurement has reduced the importance that joint stiffness plays as an RA identifier. The research areas of medical rehabilitation and clinical assessment indicate high impact applications for wearable sensing devices. As a result, the overall aim of this research is to review current sensor technologies that could be used to measure an individual’s RA severity. Other research teams within RA have previously developed objective measuring devices to assess the physical symptoms of hand steadiness through to joint stiffness. Unfamiliar physical effects of these sensory devices restricted their introduction into clinical practice. This paper provides an updated review among the sensor and glove types proposed in the literature to assist with the diagnosis and rehabilitation activities of RA. Consequently, the main goal of this paper is to review contact systems and to outline their potentialities and limitations. Considerable attention has been paid to gloved based devices as they have been extensively researched for medical practice in recent years. Such technologies are reviewed to determine whether they are suitable measuring tools.

## 1. Introduction

The sense of touch allows us to interact with the environment effectively and efficiently. Our hands are a complex structure in the human body that helps us move through a vast amount of activities in our daily lives. Likewise, the muscles and joints in the hand and forearm allows us to move with great range and high precision [1]. Additionally, thanks to its structure, humans can perform a wide range of tasks, such as grasp and lift objects as well as guide a fine thread through a small eye of a needle [2]. Sadly, hand function damage induced by diseases such as rheumatoid arthritis (RA) and Parkinson’s disease (PD) and other neurological conditions have a detrimental effect on the quality of life of the people affected [3]. Physical exercise therapies have primary clinical significance for improving motion recovery [4]. Therefore, wearable devices should simultaneously monitor all finger movements dynamically to assist with the diagnosis process of the diseases at early onset stages, and extract objective measurements of slight improvements in the hand and limb mobility during rehabilitation activities [5]. Similarly, wearable technologies (smart gloves) ought to continuously monitor the hands without being a nuisance to a patient’s daily activities [6].

This paper has the goal to provide a review of contact systems for medical applications and to provide state-of-the-art research guidance in smart wearable systems in the clinical field. The paper is organized into three main sections: an overview of the problem, a discussion on the background around RA and also diagnosis/rehabilitative methods reviewed. This leads into the second section, where various sensor technologies and other key features linked to sensory gloves is presented. The third section discusses the history behind data gloves from the early 1970s, but more importantly, reviews current state-of-the-art solutions that could be used in the clinical practice for hand functional assessment. Here, an extensive overview of the sensor positions and joints monitored on each device is discussed. Other key features, including sensor holding materials and sensor/device calibration, is also reported.

### 1.1. Problem Background

In medical applications, such as hand function assessment and rehabilitation, capturing hand kinematics is necessary [5]. RA is an autoimmune disease that mistakenly attacks multiple joints causing persistent pain, swelling and stiffness [7]. Inflammation causes the joints to degenerate, which leads to disabilities, deformities and progressive joint damage that cannot be reversed [1].

**Figure 1:**
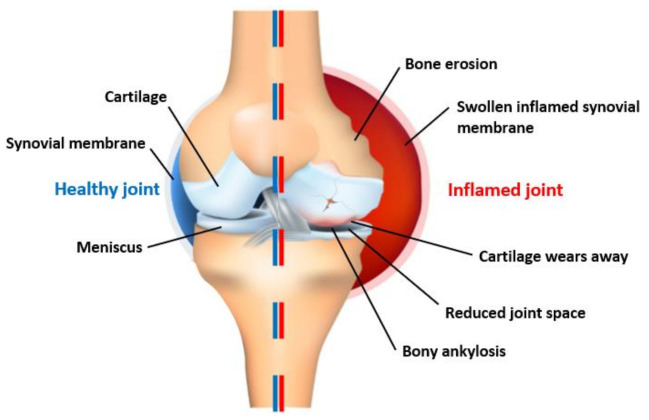
Rheumatoid arthritis (RA) joint breakdown.

The resulting inflammation seen in Figure 1 causes the synovial membrane, which lines the inside of the joints to thicken [8].

Early presentation of RA can be recognized as the appearance of an asymmetrical form of arthritis [9]. Symmetry is the key determent for the diagnosis of the autoimmune condition [10]. However, at the onset of the disease, a person may not have symptoms on both sides of the joint making it difficult to diagnose. As the disease progresses, it will only become symmetrical [11]. A sufferer with RA uses their hands to interact with the environment each day for a huge number of complex tasks and RA is the most destructive in the small joints of the hands [12,13]. Consequently, the loss of hand function caused by RA has a detrimental effect on the quality of life of those affected [14]. Therefore, intense research and clinical work has focused on the understanding of the initial onset and symptom regulation as the disease progresses. Rheumatologists need to reliably control the disease of an RA sufferer, where rehabilitation exercises can be monitored daily to reduce the disease progression rate and to move towards remission [10].

Currently there is no cure for RA, but it is commonly treated using disease-modifying antirheumatic drugs (DMARDs) and physical therapy exercises that reduce the disease progression, whilst improving overall long-term prognosis [15,16]. Regardless of this improvement, RA has a major impact on a person’s activities of daily living (ADL) [17]. There is no single test that confirms the presence of RA, although there are several RA diagnosis measures to test grip, strength, range of motion (ROM), dexterity, hand pain, hand swelling and finger stiffness [18]. There are several validated assessment methods used by clinicians to investigate symptoms of RA, such as the health assessment questionnaire (HAQ) and the ‘disease activity score 28’ (DAS28) [19,20,21]. X-rays (radiographs) are a suitable outcome measure in patients with RA, although in the early stages of the disease, X-rays may appear normal although the disease activity is high [8]. Grip strength, pinch strength and ROM are the most used outcome measures for planning treatment of RA [13,16,18,22]. Correspondingly, capturing hand functionality is necessary in clinical settings for hand evaluation and rehabilitation.

**Figure 2:**
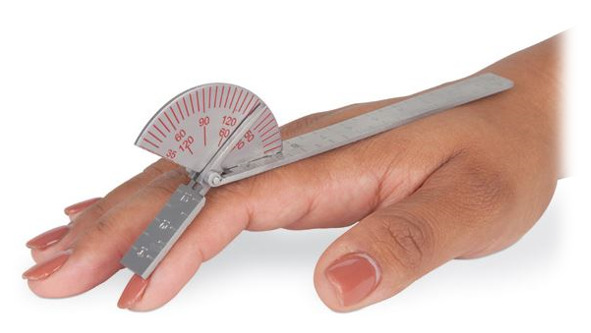
Universal goniometer (UG) [24].

ROM is the amount of measured movement around a specific joint within the body and is commonly measured with a device known as the universal goniometer (UG). A UG shown in Figure 2 is a two-armed, metal or plastic handheld device containing a gauge that represents an angular number much like a protractor [23]. 

**Figure 3:**
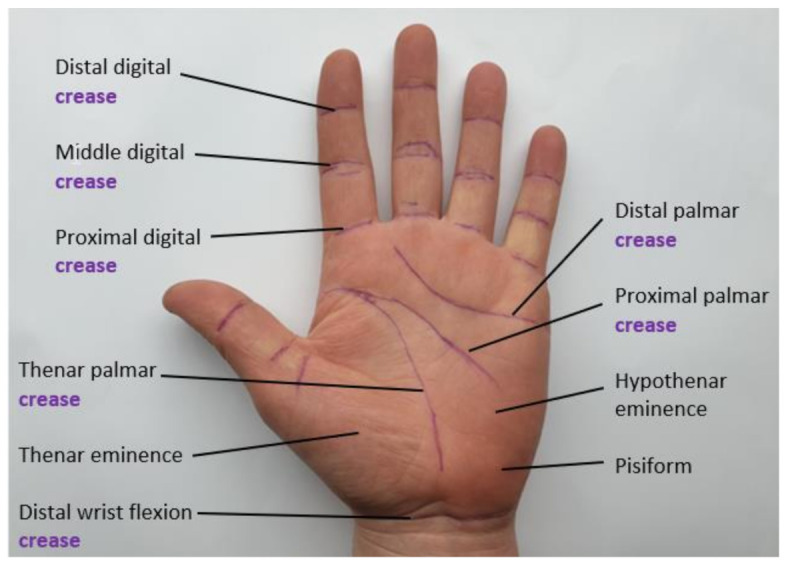
Landmarks of the palmar hand.

Physical therapists align the arms of the UG around the joint, and then shift the body in a direction to calculate the amount of motion that occurs [23,25]. These simple devices assist in the diagnosis of RA and physical therapy techniques. However, the devices give little or zero improvements in self-management activities as they require a clinician to perform measurements of the patient’s joints [26]. More generally, these two-armed devices still present several limitations even within the clinical practice. Certain factors between clinicians (inter-rater reliability) such as the positioning of the device, the therapist’s own technique (intra-rater reliability) in the interpolation of anatomical landmarks seen in Figure 3 can also cause a lack of consistency when using a UG device [27]. It follows that the issues found with the usage of the UG in the evaluation of static joint ROM can be further worsened by novice practitioners who have little experience in accurately identifying anatomical landmarks. In addition, the reliably of both the intra-observer and inter-observer is debatable. Special training is set to follow standardization through group agreement, which improves the clinical measures in Rheumatology [27].

In the discussion of Keogh et al. [28], researchers confirm that the UG has been a commonly used tool for measuring joint ROM in clinical practice due to its low cost and ease of use; dependability and validity has been established in numerous studies. Their findings suggest that the evolution of technology provides researchers and clinicians with smart devices along with complex applications with more measurement options than previously available. However, the durability and reliability of these devices and applications remains somewhat unclear [18]. 

Any measurements that are used for planning clinical care must be accurate and reliable [18,29]. There has been a great deal of research conducted using various instruments to investigate the reliability of goniometry [23]. In an earlier report, Salter [30] emphasized the importance of a standardized procedure for use of UGs as they found that the inaccuracy between clinicians is due to the lack of consistency using the device. In addition, the positioning of UGs involves four factors: the patient, the joint, UG and the clinician [23]. Salter believed that a device designed to calculate the magnitude of a joint angle should not interfere with joint motion. Hence, to improve the validity and reliability of the device, a UG requires special attention during the assessment of each joint. These findings are congruent with Milanese et al. [23] who detailed that intra and inter-tester reliability of a UG between clinicians can affect the overall outcome of the observation.

Burr [31] carried out an excessive study to establish if the inter-rater and intra-rater reliability of measurement differs between a team of therapists when measuring the finger joints using a hand-held UG. Their findings demonstrated the value of one therapist to assess the same individual and how other clinicians may be constantly low or high in their readings. Interestingly, their report also found that the therapists were usually more accurate with the UG device than 75% of the regular staff workers. Other researchers Ellis and Bruton [32] found that a skilled observer varied 7–9 degrees or less than 95% of the time whilst using a UG. In addition, the universal UG has certain drawbacks, even when assessing static movement [28].

Like joint pain, joint stiffness gets worse in the morning or after a time of inactivity. Morning stiffness which is a symptom of another form of arthritis called osteoarthritis normally wears off within 30 min of waking up, but morning stiffness in RA sometimes persists longer than that [10]. Impartial measurement of morning/joint stiffness in a clinical practice is not assessed, despite its importance given the repeated occurrence of the symptoms [7]. Connolly’s [22] findings indicate that early morning stiffness is generally reported by the patient. However, their estimated pain can be confused with other unexplained pain caused by non-inflammatory conditions and depression adding to under or over prescribing medication. Several researchers acknowledged the fact that RA affects the manipulation of objects as grip strength can be dramatically reduced [13,33]. Object handling with a comfortable handgrip is one of the most common gestures in everyday life and occupational practices. Hence, the reduction in grip strength dramatically affects a person’s ADL leaving them with a long-term disability [34].

Many patients with RA have more common effects as well as complications with the joints, such as tiredness, lack of energy, lack of strength, high fever, nausea, loss of appetite and weight loss [35]. Inflammation that is characteristic of RA may often cause complications in other parts of the body, such as dry eyes and chest discomfort [36]. Bakir, Samancioglu and Gursoy [37] have carried out an extensive study on RA patients’ pain and how it effects their sleep quality. Patients complain of exhaustion not only because of their illness, but also because of insufficient sleep. Their study discussed non-pharmacological approaches such as reflexology, massage, physiotherapy, gentle touch, acupuncture and music therapy been used in RA patients to monitor and enhance their functioning reducing the need for aggressive DMARDs. However, whilst their study on the effect of therapy on pain symptoms has been measured in patients with RA, no research on sleep quality has been undertaken. Researchers Kerschan-Schindl and MacHold [34] found that various physical symptoms contributed to inactivity including discomfort, swelling, injury to the joint, low bone density and muscle fatigue. Their study of physical activity for RA also found that various physical symptoms may cause the patient to stop exercising if they aggravate their condition. Metsios and Kitas’s [38] recent study specifies how inactivity of the affected joints decreases ROM and reduces the ability to perform the movements needed for ADL. Their findings indicate that patients with RA are typically directed to complete therapy with low impact activity to maintain cognitive ability and to enhance their functional capability.

Several researchers have recognized the importance of hand dexterity and how patients can benefit by completing daily hand exercises. Specifically, Smolen et al. [15] recognized that physicians can reliably evaluate the condition by monitoring and recording hand functions on a daily basis. Similarly, the study carried out by Majithia and Geraci [10] discussed how it is vital to diagnose RA at early stages to prevent the development of joint erosion. Therefore, clinicians seek a reliable and practical device to replace traditional methods that could assist in the diagnosis measures of RA at early stages. Given the above, a realistic and accurate device that can monitor many parameters of hand function assessment and rehabilitation activities is therefore necessary.

### 1.2. Hand-Based Functional Assessment

Hand-based functional assessment is used to assist in the diagnosis of RA and other neurological conditions to start effective treatment [39]. Clinical applications and in particular, rehabilitation activities are mainly concerned with hand-based functional assessment [7,25,33,40]. To clarify, the assessment of hand functionality for clinical purposes requires the gathering of several data forms, including for example, resting state, grip strength, velocity, acceleration, and hand joint ROM, etc. Several researchers have conducted investigations into contact systems [22,25,41,42,43,44,45,46,47]. However, current research seems to indicate that contact systems are more sought-after for monitoring hand functionality [1,5,9,29,48,49]. Several types of sensors have been used to measure the quantitative measurements of interest, with complex architectural and circuit designs. Solutions offered by wearable devices eliminate the inter-tester and intra-tester reliability issues that originate with the UG. In addition, the devices have the capability of increasing the accuracy and repeatability measures within the clinic, whilst enhancing the complexity of measurements due to the continuous operation of multiple sensors.

**Figure 4:**
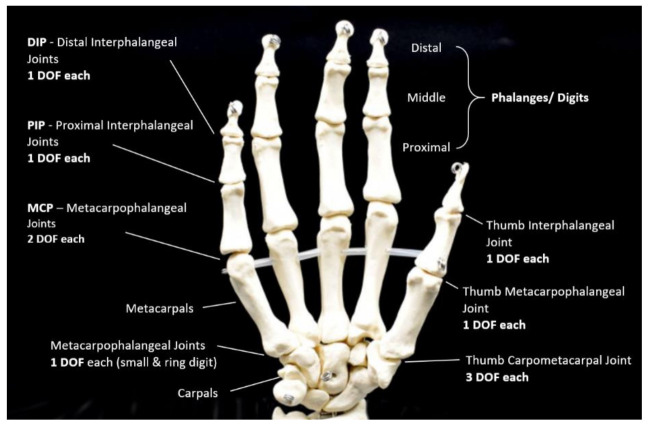
Degrees of freedom (DOF) of hand joints.

The human hand comprises 27 bones of approximately 25 degrees of freedom (DOF) guided by 17 inner muscles in the hand and 18 outer muscles in the forearm [44]. Consequently, the grasping hand is a valuable source of inspiration for engineers and scientists to develop human-like robotic and prosthetic hands and to research and improve human hand function capabilities. Several researchers have focused mainly on assessing the ROM on the fingers and thumb. Reports by O’Flynn et al. [48] have found that Rheumatologists specifically measure the metacarpophalangeal (MCP) joints, proximal interphalangeal (PIP) joints and the distal interphalangeal (DIP) joints of the fingers. Additionally, their study also measured the thumb, which contains the carpometacarpal (CMC) joint, MCP joint and an interphalangeal (IP) joint. The study by Ruffing [50] indicates that RA often appears in one or more of the MCP and PIP joints. Anuj P Netto [51] claimed that the DIP joints are affected less frequently. However, if they are affected, it is typically only after RA symptoms of the MCP and PIP joints. While osteoarthritis is the most prevalent cause of thumb arthritis, RA can often impact the CMC joint, typically to a lesser degree than the finger joints [51]. Figure 4 demonstrates the DOF of each of the hand joints.

The CMC joint of the thumb has three DOFs, unlike other joints, whilst the MCP joints have two DOF, the PIP and the DIP joints having just one DOF [33]. Clinicians and researchers have recognized the importance of hand kinematics to benefit the assessment criteria needed for diseases such as RA [52]. This includes accurate measurement of flexion, extension, adduction, abduction of all finger and thumb joints, including the complex web space movement measurement of the CMC joint [41]. The ROM is the most commonly useful way of monitoring the functionality of the hand for RA measures [52]. Clinicians and researchers have established tools and systems to benefit this process.

Hand monitoring systems have been used for years throughout human computer interaction (HCI) [44]. However, these technologies have only been used in very standardized conditions such as research centers and experimental laboratories for medical use. To be valuable and broadly usable in clinical settings, devices must be precise and accurate within the conditions of everyday living [53]. Likewise, their physical structure must be robust towards providing the desired results with the variations in hand sizes. These factors have often been previously discussed as disruptive for complex hand monitoring systems used in the medical field. Furthermore, these challenges are an important attribute to data glove technologies because participation in physical activity is recommended for rehabilitation without complications, resulting in both psychological and clinical benefits with improvements in ROM function [53]. Currently, several data gloves have been developed for monitoring patients’ physical activity. They have proved valuable for monitoring ROM and for detecting basic hand gestures [53,54].

In conclusion, with the correct combination and situation of sensors on joints of interest, an adoptable glove material/exoskeletal device, a system for gathering and monitoring the data, and it all can be used to assist with the early onset of the disease and the end physical activity in RA [5].

## 2. Sensors Characteristics & Signal Processing & Output

Sensor gloves have been widely researched in applications such as robotics and VR, and they have gained increased significance in recent decades for medical applications [49]. This paper emphasizes the importance of research in sensor glove devices for medical and health care domains. Research evidence proposes that sensory data gloves are the state-of-the-art technology for RA goniometric measurement and for the rehabilitation of diseases requiring quantification of finger joint flexibility [48]. Even though sensor gloves are relevant in the assessment of hand dexterity, their medical use is limited by a number of issues, including accuracy, repeatability, and connectivity to internet of things (IoTs) [44], as well as their usability and comfort [22].

### 2.1. Sensor Topologies

There are promising opportunities for data gloves to assist in RA diagnosis and rehabilitation measures, and monitoring of other mobility diseases within the health care environment [43]. The sensor is one of the core data glove technologies that provides measurement of hand dexterity, and is capable of measuring bend, motion, rotation, and hand position [55].

The most significant parameters for hand rehabilitation are the measured angles of the joints [40,52,56,57]. Likewise, a variety of movements are provided by synovial joints in the hand [48]. The contraction or relaxation of the muscles that are connected to the bones on either side of the articulation results in each motion at a synovial joint [58]. Flexion and extension movements of the MCP, PIP and DIP occur in the sagittal (anterior-posterior) plane of motion. Abduction and adduction of the digits occur on the coronal (medial-lateral) plane of motion [22,58]. Correspondingly, the sensors generally used to monitor hand function belong to the following categories.

#### 2.1.1. Sensors Used to Monitor Finger ROM

The most prominent action that can be accomplished by all digits (little, ring, middle, index finger and thumb) is to bend them to the palm (flexion) and then back (extension) to the original position. The thumb has unique advantages over all other fingers with a complex CMC joint that has three DOF allowing it to move freely in six DOF counting the other two joints [33]. In particular, the prevailing movement required to detect stiffness and to assist RA diagnosis and rehabilitation is related to the bending of all digits [7]. Finger bend can be observed using a variety of sensor technologies, as seen in the literature.

**Figure 5:**
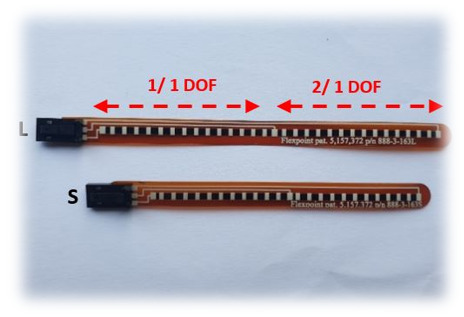
Flexpoint 2-in-1 sensor.

**Figure 6:**
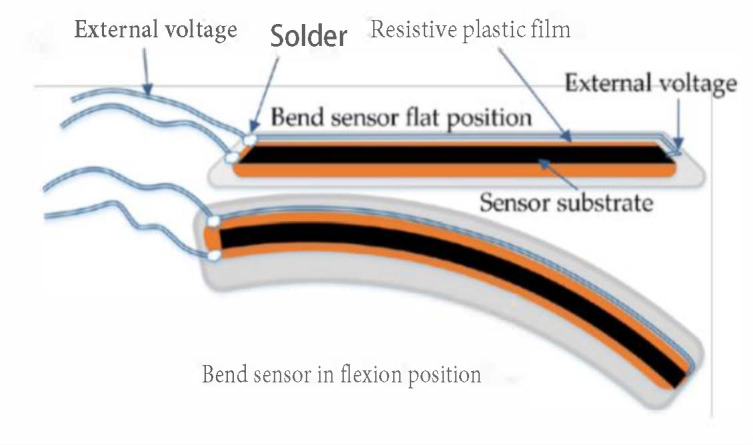
Flex sensor characteristics [22].

The most popular sensor used in a data glove is a flex sensor (resistant transducers). This type of sensor measures the degree to which the sensor curve deviates from a straight line, and is commonly used by researchers and engineers [9,41,52]. Resistant transducers are lightweight, low cost, and vary in size to suit applications [59]. The flex sensor is therefore quite easy to implement within existing technologies, and can be customized for varying user requirement [60,61]. Resistant flex sensors have increasingly been used in various cases [62]. Similarly, static and dynamic postures can be documented in medical applications when used in a glove device [29]. Given their interesting properties, such as robustness, low price and long life, they frequently show non-linear response and lower sensitivity, particularly at small bending angles [5,22]. Nevertheless, a research clinical glove developed by ActionSense [60] uses multiple Flexpoint bend sensors [61] to monitor hand function. Figure 5 shows two custom 2-in-1 combined flex sensors (two sizes, short 5” and long 6”) that have two outputs to monitor two finger joints on one sensor. The internal flexible layer (seen in Figure 6) protected by carbon-ink measures the difference in resistance to the bending angle of the transducer [44,61].

**Figure 7:**
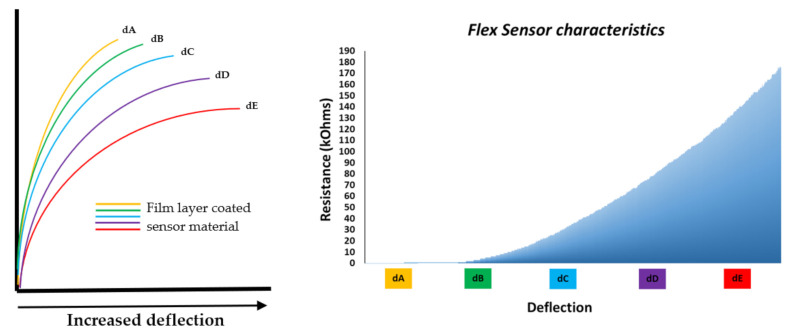
Flexpoint deflection.

As shown in Figure 7, the substrate is bent from position dA to dE. The resistance performance of the sensor is proportional with the bend radius as the smaller the radius, the higher the resistance value. Likewise, the flatter the sensor substrate is, the lower the resistance (nominal resistance). As the angular position reaches 45 degrees, the sensor resistance rises to twice the nominal resistance. Additionally, as the sensor is bent to 90 degrees, its output resistance can increase to four times the nominal resistance [63].

**Figure 8:**
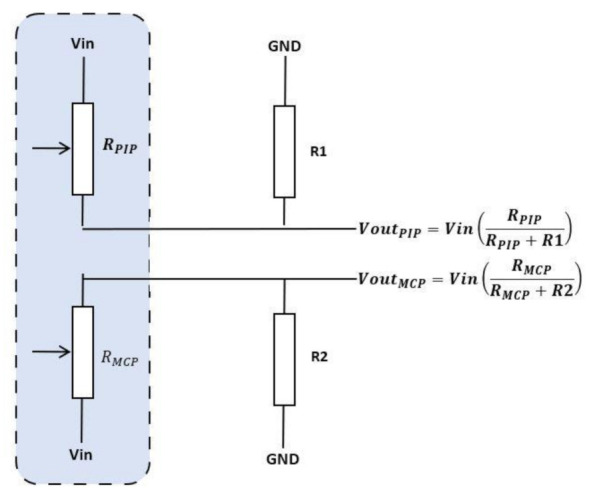
Custom 2-in-1 combined Flexpoint sensor with voltage divider circuits.

The flex sensor is usually connected to a voltage divider circuit, shown in Figure 8. The illustration presents two sensors incorporated into one Flexpoint sensor, where an electrical potential at the output of the flex sensor is collected by an analog-to-digital converter (ADC) circuit and then sent to the processing unit where it is monitored by a controlling algorithm. Generally the flex sensor uses a buffer (op-amp) circuit to enhance/amplify the signal at the output of the sensor (not in illustration) [44].

**Figure 9:**
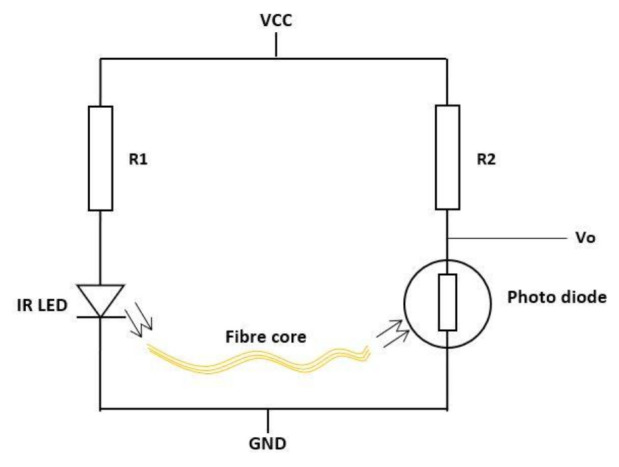
Fiber optic circuit.

**Figure 10:**
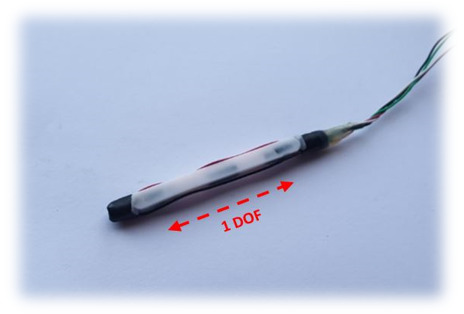
Fiber optic sensor.

An optical sensor uses the characteristics of optical technology to determine the curvature angle of a bend [64]. The sensor measures infrared (IR) light emitted from a diode transmitted through a fiber core to a detector (photo diode) [64,65], as shown in Figure 9. When the fiber core is bent, the photo diode experiences changes in the received light within the signal processing circuitry (Vo). Fibre optic sensors have conventionally been used as a luminescent source that is transmitted via an optical fiber to a light dependent resistor. Both optical technologies operate on the measurement of light intensity so that when the sensor is flat, as representative of a “flat hand” position in a data glove, the intensity of the light measured by the Vo will be significant. Additionally, the inverse will be true once the sensor becomes bent [66]. Optical fibers are lightweight, flexible, reliable, and immune to electro-magnetic interferences, making them well suited for wearable devices [44,56,58,67,68]. Pasquale’s [44] findings suggest that optical fiber sensors are well suited for application in cutting-edge wearable systems. However, additional stresses induced into the fibers whilst placed on a textile substrate can cause them to be sensitive, causing light attenuation resulting in sensor inaccuracy [44,66]. The 5DT (Fifth Dimension Technologies) commercial glove [69] has 14 unique fiber optic bend sensors presented in Figure 10. For instance, each sensor is strategically placed inside individual small pockets of a textile glove that monitor all the digits except for the DIP joints of the fingers and the CMC joint of the thumb [41]. Similar to the flex bend sensors, they do not have the ability to detect hand orientation with only one DOF available between each sensor.

**Figure 11:**
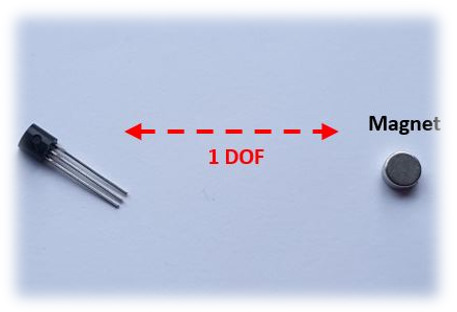
(SS495B) Hall effect sensor.

Hall effect sensors are based on the variation of the output voltage in response to a magnetic field [45]. The SS490 series are lightweight, flexible, linear devices powered by a magnetic field from a permanent magnet or an electromagnet [70], as shown in Figure 11. The Hall Effect sensor (SS495B) responds to either positive or negative Gauss (north and south poles) [71]. Moreover, these sensors have been previously placed on the tips of the fingers with a magnet on the palm of the hand [59]. Changes in the proximities of each sensor to the magnet vary the output voltage. There is little evidence of Hall effect technologies being used in commercial or research data gloves except for the Human Glove [25].

**Figure 12:**
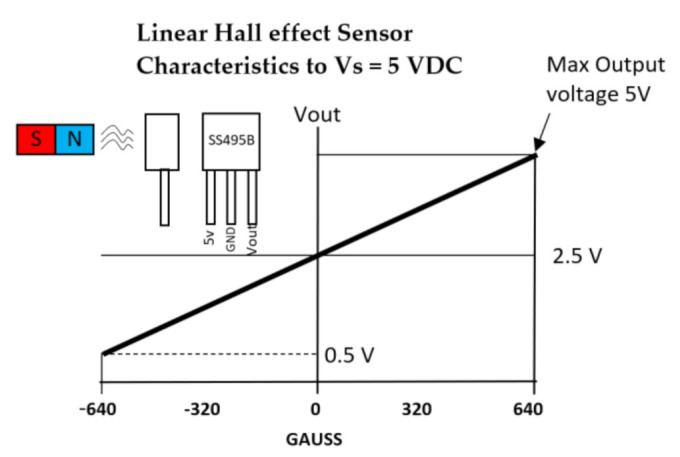
Hall effect characteristics at max 5 V.

Hall effect sensors are characterized as analogue or digital, depending on their feedback signal. In an analogue sensor, the feedback signal is of a constant type and is directly proportional to the force of an applied magnetic field [1]. In addition, the increase in the force of the applied magnetic field increases the resultant output voltage until it is saturated by the restriction applied to it by a power source [71,72], as shown in Figure 12. Likewise, a digital sensor acts like a switch; if the magnetic field reaches a predetermined value, the output of the sensor changes from “OFF to “ON” [1]. Hall effect sensors are usually integrated into an exoskeleton device similar to the early Human data glove [25,40]. Owing to their small size and the concept of contactless operation, these sensors allow the device to operate smoothly, preventing frictional resistance [72]. A main disadvantage of this technology is that they cannot sufficiently provide accurate orientation information, since the sensors measure only one DOF of the joint [73].

**Figure 13:**
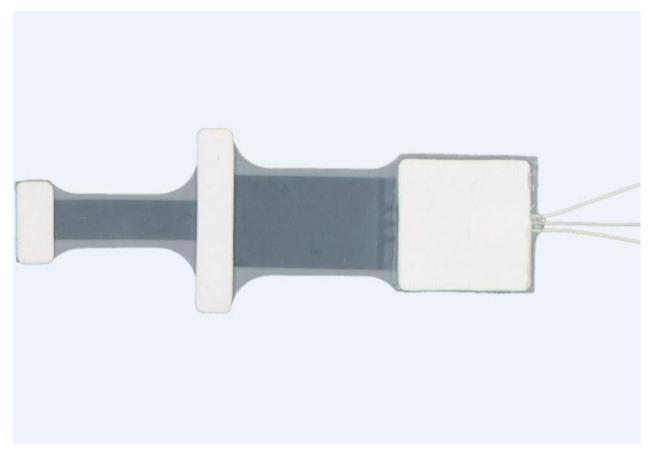
StretchSense capacitive sensor [74].

Capacitive Bend Sensors are not commonly used in research or commercial gloves. However, the manufacture StretchSense (Auckland, New Zealand) is the first to commercialize a product with capacitive technology with their most recent uniquely designed 3-in-1 sensor [74], shown in Figure 13. 

**Figure 14:**
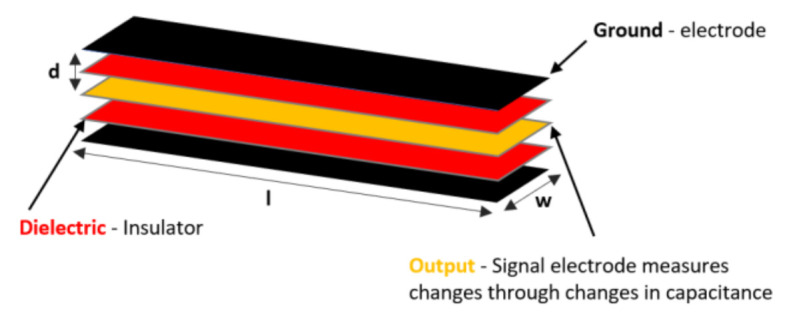
Parallel plate capacitor model of the pressure sensor.

Their MoCap Pro SuperSplay glove device is also seen in Section 3. Their stretch sensors are flexible capacitors that can measure the stretch, bend, shear, or strain. Moreover, the sensors capacitance values change as the sensor deforms (due to stretching or squeezing). Depending on the desired use, StretchSense sensors may be developed for various sizes, with varying amounts of elasticity and sensitivity [74,75,76]. The internal sensors consist of a stretchable signal electrode placed between two ground electrodes, which are separated by dielectric silicone insulators, as shown in Figure 14. The sensor is a versatile parallel plate capacitor, and the thickness and area of the conductive substrate changes as the sensor is extended. Stretching the sensor increases the surface area, and reduces the sensors thickness, leading to an increase in capacitance [76,77].

The capacitance output (farad) for a parallel plate capacitor is described by:

$$C=\;\varepsilon_{r}\varepsilon_{0}\frac{lw}{d}$$

Since the capacitors are resistant to a change in voltage, the capacitance can be determined by applying a small voltage. Thus, the capacitance of the sensor can be measured by adding a voltage to the sensor and comparing the real voltage response to that expected without a capacitor [76].

**Figure 15:**
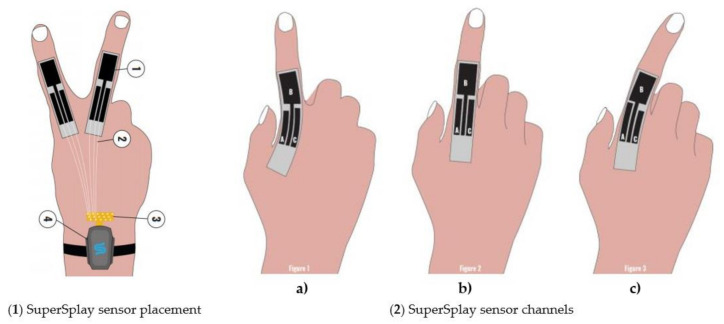
StretchSense sensor illustration [74].

Figure 15 is an illustration (1) of a SuperSplay sensor that is attached to both the index and the middle finger. In the illustrations (2(a, b, c,)) is a splay sensor with 3 segment channels; A, B and C with the index finger in different positions. Channels A and C of the sensor determine the splay and MCP bend movement of the finger. Channel B is placed or stretched above and between channels A and C to capture 1 DOF bending of the PIP joint. In illustration (a), channel A decreases (compresses) in length and channel C increases (stretches) in length. Equally, when the finger moves to the right, channel A increases (stretches) in length and channel C decreases (compresses) in length, as shown in illustration (c). Examining these differences (one minus the other) measures the splay direction of the finger. With varying lengths and thicknesses, the capacitance values of each channel differ. Illustration (b) channel A and channel C are the same length, demonstrating that the MCP is in its normal position (finger straight). Finally, the MCP joint is monitored by both segments (channel A and channel C) under stretch. Both values increase, and the MCP bend is therefore related to the sum of both sensor segments [74].

#### 2.1.2. Sensors Used to Monitor Finger ROM and Hand Orientation

**Figure 16:**
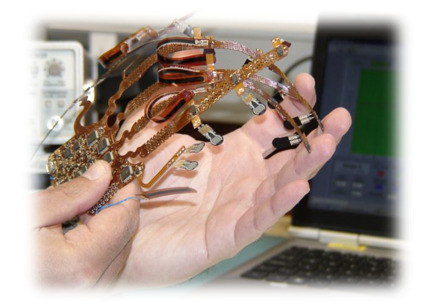
UU/Tyndall accelerometers [41].

Despite the benefits of using the aforementioned sensors to determine finger bend, hand orientation cannot be distinguished by such sensors. The characteristics of an accelerometer sensor make it possible to discern the acceleration and rotation of the hand as a further consideration in addition to its ability to determine the bend of the finger [44]. Researchers, O’Flynn et al. [41], added several accelerometers (ADXL345 Adafruit Industries [78]) to their device to improve the accuracy of both static and dynamic finger joint movements, seen in Figure 16. Likewise, the bend sensors work in collaboration with accelerometers to precisely measure the angle, velocity and acceleration of the finger joints. Thus, the same three-axis accelerometer, which supplies variations in acceleration along each joint is also placed on the palm of the hand. This captures the orientation and movement of the wrist to accurately represent the essential feature of a sensor glove [41].

**Figure 17:**
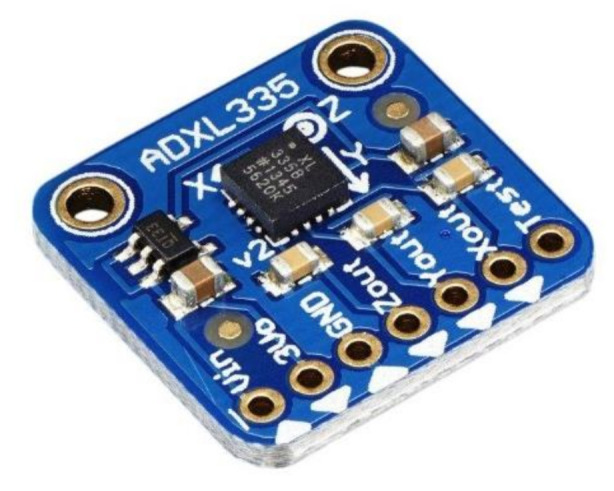
Adafruit ADXL335 (Breakout Board).

A similar device (ADXL335 Adafruit Industries [79]) is shown in Figure 17; it is a thin, small, low powered 3-axis accelerometer with signal-conditioned voltage output. The device measures acceleration with a full-scale minimum range of ±3 g. In tilt-sensing applications, it may calculate the static acceleration of gravity, as well as dynamic acceleration generated from motion, shock, or vibration [79]. It includes signal-conditioning circuitry for the implementation of an open loop acceleration of the measurement architecture. The output analog voltages are proportional to acceleration [59].

The sensor is a surface-micromachined polysilicon structure mounted on top of a silicon wafer. Polysilicon springs suspend the frame above the wafer surface and are resistant to acceleration forces. Deflection of the structure is measured as a differential capacitor composed of individual fixed plates and plates connected to the moving mass. Acceleration deflects the traveling mass and unbalances the differential capacitor, resulting in the output of the sensor, the amplitude of which is equal to the acceleration. In contrast, to determine the magnitude and direction of the acceleration, phase-sensitive demodulation techniques are then used [79].

**Figure 18:**
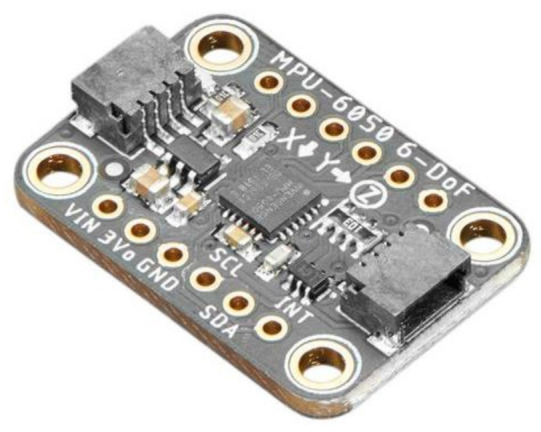
Adafruit MPU-6050 (Breakout Board).

The (MPU-6050 Adafruit Industries [80]) sensor shown in Figure 18 was initially used for smartphones, tablets and wearable sensors with low capacity, low expense, and high-performance specifications. Additionally, the sensor integrates InvenSense MotionFusion and the run-time configuration firmware, allowing manufacturers to avoid expensive and complex collection, qualification and system level integration of discrete devices in motion-enabled devices, ensuring that the fusion algorithms and calibration processes of the sensor provide optimum output [81]. 

The sensor systems incorporate a 3-axis gyroscope and a 3-axis accelerometer with an onboard Digital Motion Processor running sophisticated 6-axis MotionFusion algorithms on the same silicon chip [80]. The 6 DOF unit provides data on accelerations in all three directions, plus angular rotation around each axis [59]. The MPU consists of three different vibratory MEMS rate gyroscopes that detect X-, Y-, and Z- axis rotation. A Coriolis effect induces a vibration detected by a capacitive pickoff when the gyros are rotated along either of the sensory axes [22]. As a result, a voltage is generated that is proportional to the angular velocity; the resulting signal is amplified, demodulated, and filtered. This voltage is then digitized to sample each axis using individual on-chip 16-bit ADC [80]. The gyroscope is intended to determine the angular orientation based on the concept of spatial rigidity. Correspondingly, an accelerometer cannot determine the precise orientation of an object in motion. As a result, gyroscopes measure angular velocity and are not influenced by gravity [59].

**Figure 19:**
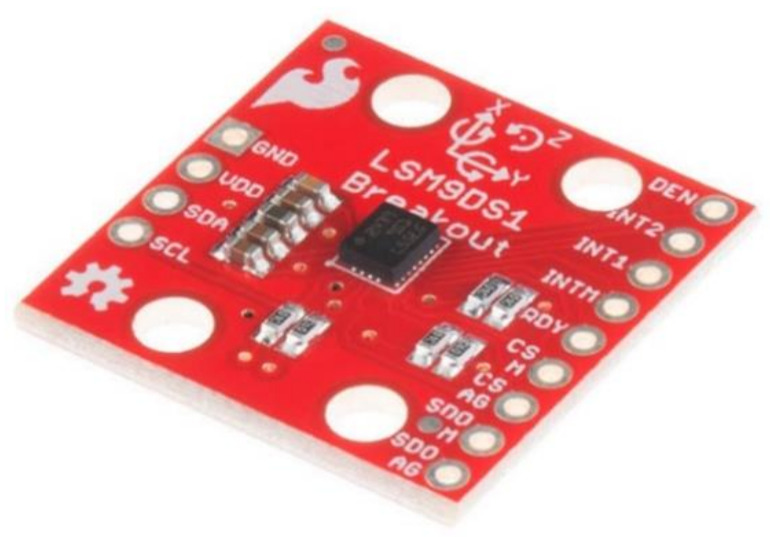
SparkFun LSM9DS1 (Breakout Board).

**Figure 20:**
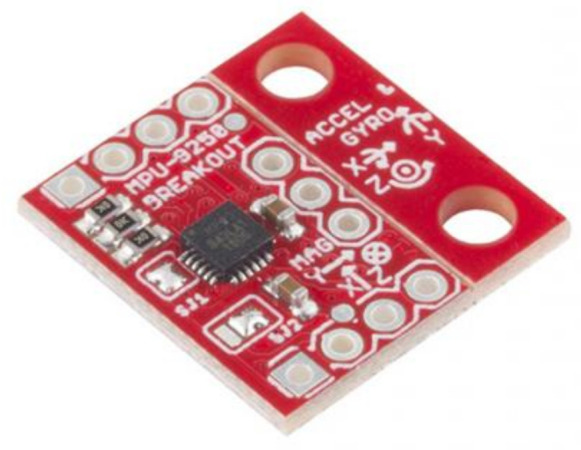
SparkFun MPU-9250 (Breakout Board).

A nine-axis inertial measurement unit (IMU) sensor can be obtained by connecting a three-axis magnetometer to a three-axis accelerometer and a three-axis gyroscope. However, a IMU device generally has the three-axis gyroscope, three-axis accelerometer and the three-axis magnetometer all combined within one integrated chip [6]. Two popular IMU sensors (LSM9DS1 [82] and MPU-9250 [83]) are shown in Figure 19 and Figure 20. 

These devices are very popular within the clinical environment [57]. Hence, they can monitor hand kinematics precisely, whilst providing useful data such as acceleration, angular velocity, magnetic field and ROM as manual dexterity parameters for physicians [5,29]. Furthermore, the devices are the most practical for wearable sensing gloves, since they are lightweight, small and can accurately measure minor changes in finger movement [5]. The small electronic devices provide nine DOF capturing nine distinct types of motion or orientation related data [48]. These devices, however, require complex programming, it can be costly, and require shielding from magnetic interferences [5,29,49]. The MPU-9250 contains a three-axis magnetometer that utilizes Hall sensor technology that is extremely sensitive. The magnetometer component of the IC contains magnetic sensors, a sensor driving circuit, a signal amplification chain, and an arithmetic circuit to detect terrestrial magnetism in the X-, Y-, and Z-axes to process the signal from each sensor [83].

In addition, both devices use either an inter-integrated circuit (I2C) or a serial peripheral interface (SPI), serial communication protocol to connect with a system and often function as a slave when communicating with the processor. A comparison of the aforementioned sensor technologies is listed in Table 1.

### 2.2. Microcontroller/Processing Unit

The processing of a sensors signal is aimed at optimizing the raw signal coming from sensors using effective filtering techniques. Depending on the sensor output, the signal may still be raw and thus require additional electronic processing before being processed by a microcontroller. A signal conditioning circuit consisting only of resistors and operational amplifiers can be used with flex sensors to achieve a stronger linear relationship between the resistance of the sensor and the angle of bending [41,44]. Moreover, the signal coming from the conditioning (analog) circuit is sent to the ADC then to the microcontroller. On the microcontroller, a developed program converts the measured inputs into angles and transmits them to a host computer or an output display [5,25,29,41,42,49]. Various digital sensors also offer onboard ADC convertors and no external conditioning circuits are required [22,33,41,44,84]. SPI, I2C and universal asynchronous receiver-transmitter (UART) are the most used protocols commonly used in microcontroller systems [29,43].

The microcontroller is the brain of the device responsible for collecting data from all the sensors and for carrying out the necessary processing in order to identify and pass the signal to the output port to be displayed at the final level [59]. A high-performance Atmel AVR32 UC3C 32-bit microcontroller was selected by O’Flynn et al. [48] for their complex hand monitoring sensory device. The device has low power consumption, a 32-bit AVR microcontroller with a built-in single precision floating-point unit. Moreover, it was selected to allow for the development of complex motion-focused embedded algorithms for real-time low-energy operation. Many researchers have used development microcontroller boards [16,59,85] such as Arduino Nano, Uno, Mega as they are widely available on open source electronics platform. The ATmega328P microcontroller is found in the Arduino Uno [85] and has 14 digital inputs/outputs, 6 analog inputs, a 16 MHz quartz crystal, and a USB port [59].

### 2.3. Output Display Monitor

System developers and researchers usually attempt to communicate with sensory devices through a digital liquid-crystal display (LCD) or graphical displays. Connolly [22] implemented a graphical user interface in designing a glove-based medical system for RA. This platform offers a user-friendly interface for the end user (doctor, recovery trainer, patient, etc.) that shares information automatically. The modern mobile device is essentially another option favored for sensory system outputs. The ActionSense glove [60] uses a user friendly app with a graphical interface used to receive, display and save data for the user [9]. With technological advances, the graphical user interface provides non-specialist users with animations and visual information capable of facilitating the proper use of the whole system [44].

## 3. Commercial and Non-Commercial Glove-Based System

Throughout this section, several researchers have conducted investigations into contact systems (data gloves/smart gloves). Recent research by Pasquale [44] discusses how data gloves are used for a wide range of applications, such as virtual reality (VR) and robotics, but have gained a lot of interest in the medical field over the last decade. Their findings also indicate that sensory glove devices are most appropriate for monitoring hand movement, although it has not yet been demonstrated how accurate these devices really are. Other researchers have raised doubts on whether these devices can be suitable in medical practice [49].

Without environmental limitations, wearable devices are more realistic for accurately recording hand kinematics. Data glove systems are preferable over visual based systems as they are portable, inexpensive, and require less processing power [45]. Similarly, there is a growing trend towards smart phone usage for the measurement of joint angles [28]; such goniometry applications have recently become available, but are limited to measuring angular measurements on one surface giving only one DOF [23]. For this reason, data gloves are the most popular and researched wearable system capable of matching the hand’s complex structure [33].

Existing data gloves can be separated into two categories, commercial and research [41]. Gesture recognition glove-based systems are defined as hand-worn devices comprising an array of sensors, specific electronic circuitry for data processing, wired or wireless connectivity type (Wi-Fi, Bluetooth) and a material structure to position the sensors [16]. Rheumatologists and physicians can observe hand motions through multiple sensors attached at the joints or on the fingers of a textile or non-textile glove [56]. Likewise, sensors and electronic components are commonly stitched or mounted on a textile material that can be placed on the user’s hand to capture data relevant to hand configuration and movement [44].

With improvements in technology, a broad range of sensors have been integrated into data gloves to monitor hand kinematics, including mechanical sensors, resistive sensors, optical fibre sensors, Hall effect sensors, capacitive sensors and IMUs [1,44,56,58,74]. However, major weaknesses in practicality, power consumption and accuracy of the sensors still exist [86]. Depending on data gloves sensor types, they can have several drawbacks: they can require substantial calibration, can be difficult to fit and doff, and can be difficult to fit specific hand sizes, thus requiring small, medium and large gloves to fit all variations [48].

Sturman and Zeltzer [87] found that an Electronic Visualisation Laboratory in 1977 created the Sayre glove. The glove was based on the idea from Rich Sayres early interventions using flexible tubes mounted along each finger of the glove. A light source is attached to one end and a photocell is attached to the opposite end where the voltage of the photocell varied whilst the finger was bent. However, the glove was never used as a gesture device due to its limitations.

The Data Glove was developed in 1982 by Thomas G. Zimmerman. Zimmerman filed a patent (US Patent 4542291) on an optical flex sensor mounted in gloves for the measurement of finger bending. The device had small plastic tubes and light sensors capable of measuring and storing joint angles [45]. In later years, Zimmerman partnered with Jaron Lanier to integrate ultrasonic and magnetic hand location monitoring technologies for the generation of Power Glove and Data Glove, respectively (US Patent 4988981, filed in 1989) [44,45]. As a result, the Data Glove was a clear improvement over existing camera-based hand-monitoring systems, as it did not rely on line-of-sight observation. Furthermore, the device monitored 10 finger joints whilst being lightweight, comfortable and unobtrusive to the user. It was widely used across the world in research institutions [87]. In fact, the accuracy of the glove was said to have a one-degree resolution informally. However, official testing showed the inaccuracy to be more in the range of 10 degrees. Sturman and Zeltzer’s [87] findings indicate that the device was not suitable for accurate or complex hand gestures.

**Figure 21:**
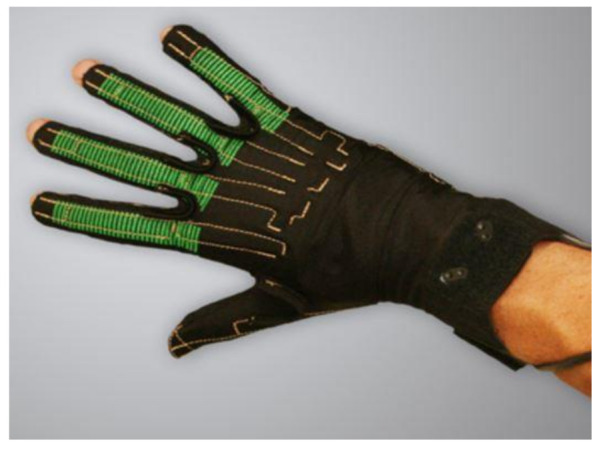
CyberGlove III [89].

Kessler, Hodges and Walker [88] carried out an extensive study of the commercialised CyberGlove model CG1801 input device created by Virtual Technologies (Palo Alto, CA, USA). In general, they conducted an experiment to investigate the level of accuracy of the sensors and the factors that affected the accuracy of the flexion measurements. Tests were completed on 18 of the 22 sensors; there were obvious differences across each of the fingers at low angles. They found poor performance of non-linearity of the sensor’s response and stated no guarantee that a joint could repeat an angle with the device’s high error rate. In 2010, a third advanced prototype of the CyberGlove was created known as the CyberGlove III [89] seen in Figure 21. To clarify, this model consists of 22 piezoresistive sensors that measure finger flexion, extension, abduction and adduction that is used for virtual modeling [44].

**Figure 22:**
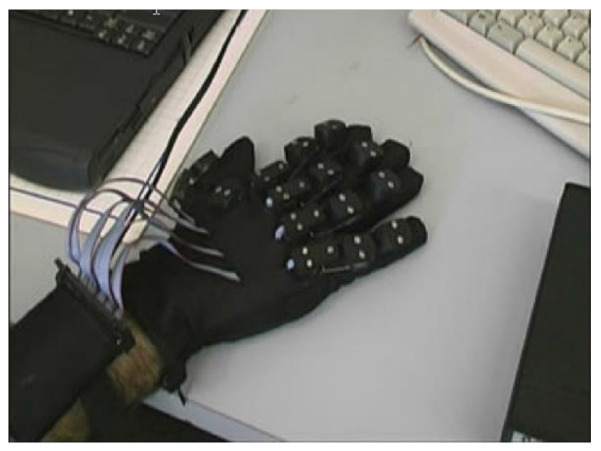
Depicts the Humanglove having 20 Hall effect sensors [1].

Figure 22 depicts the Humanglove that was fitted with 20 Hall-effect sensors that measured flexion/extension and adduction/abduction of finger joints [1]. It is known that these devices vary widely in data richness (resolution and bandwidth), device complexity and mechanical robustness [25]. However, no detailed information about the sensors is available around performance in a data glove.

Wu et al. [86] presented a non-contact glove mechanism based on electrostatic induction and triboelectric effects. They directly captured the strain or pressure information from the sensors. Their results demonstrated a novel approach for detecting one-dimensional movements and basic hand gestures. 

**Figure 23:**
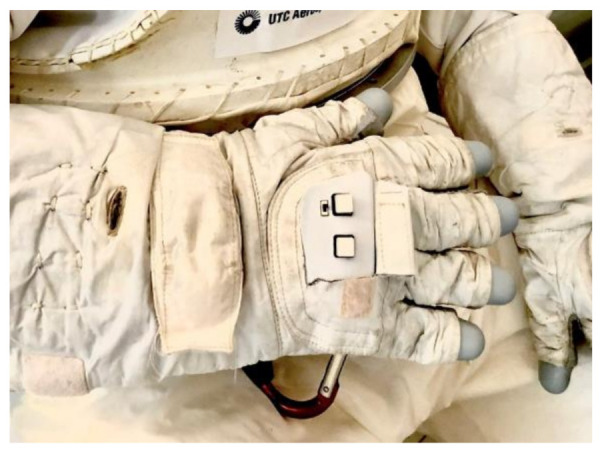
NASA (Ntention) –smart glove [90].

A number of researchers, including NASA, created data gloves in the early 1980’s to interact with the simulated airflow around an aircraft [45]. Current research from NASA and partners (Ntention) proposes a ground-breaking smart glove seen in Figure 23 for human exploration to Mars [90]. Their device uses human machine interface (HMI) to allow humans to communicate with machines using hand movements. Specifically, their glove is designed to control drones and other robots with simple hand gestures. For this reason, the smart glove proposes good finger dexterity for the wearer [45]. Furthermore, astronauts can effectively monitor a variety of robotic assets, making research and discovery activities on the Moon, Mars and other destinations more successful and profitable [90]. No information about the sensors type is available.

**Figure 24:**
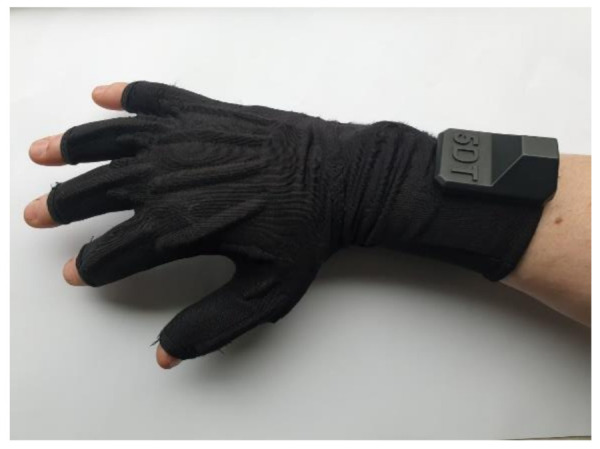
Fifth Dimensions–5DT smart glove.

The 5DT data glove seen in Figure 24 was designed by Fifth Dimension Technologies and uses 14 proprietary optical-fiber flex sensors placed in a textile substrate. The glove was designed to satisfy the stringent requirements of modern motion capture and animation professionals [91]. In addition, the sensors are placed across the MCP, PIP of the fingers and the IP, MCP of the thumb in small fabric pockets. Furthermore, the abduction and adduction movements of the thumb are monitored and between each finger using the same type of optical-fiber flex sensors placed on the same textile substrate [42]. Moreover, the CMC joint is not monitored or is the DIP joints [41]. Hence, the loose CMC capsule permits rotational movement in its unique plane, making it difficult to monitor, unlike other joints of the hand.

**Figure 25:**
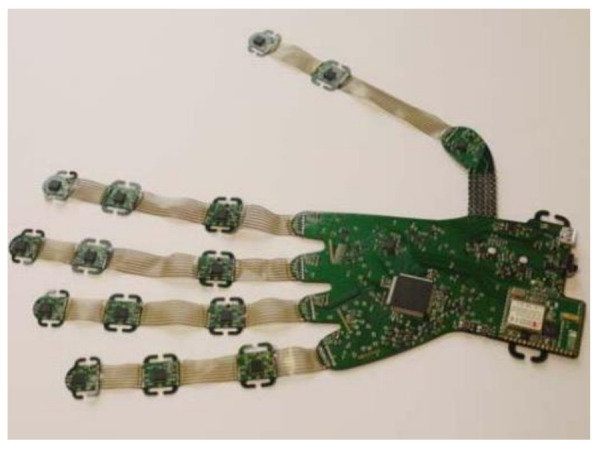
IMU Smart Glove rev 2 [68].

Many data gloves have been proposed so far using IMUs as they are small, lightweight and are one of the most realistic sensors as they can reliably record subtle changes in finger movements [49]. Recent commercialized and research data gloves have been acknowledged and examined to see if they are fit for clinical practice. O’Flynn et al. [48] carried out extensive research on modern data gloves such as the 5DT (fibre optic) and their proprietary IMU glove (Smart Glove rev 2) [68] seen in Figure 25. 

**Figure 26:**
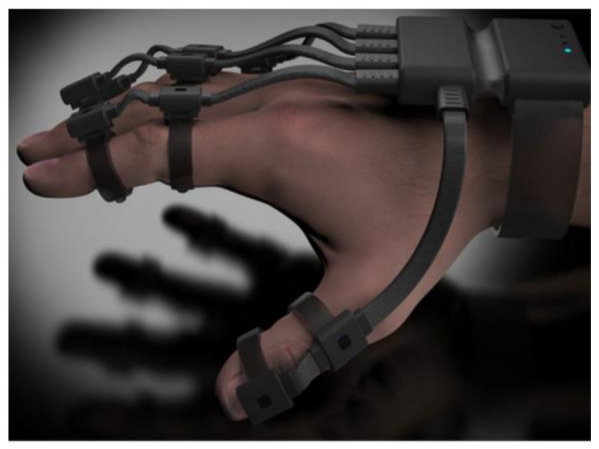
VR glove [67].

The IMU Smart Glove rev 2 has been developed using a combination of bendable and lightweight technologies. Additionally, the glove comprises 16 9-axis IMU’s strategically positioned to compensate for the DOF of each hand finger joint. To assess orientation and biomechanical parameters of each joint, IMUs are placed on the extendable interconnect and are situated on the phalange of each finger segment. Furthermore, the relative direction of each IMU is measured and used to produce angular and velocity motion during the flexion and extension exercise of each finger joint. The rev 2 smart gloves quantitatively monitor finger joint ROM, including flexion, extension, adduction and abduction of the MCP, PIP and DIP. Moreover, the thumbs CMC, MCP and IP joint are monitored together during thumb-index web spacing and palmar abduction movements [22,42]. Lastly, their rev 3 VR glove is a similar device seen in Figure 26, but does not monitor the DIP of the fingers or the CMC of the thumb [67]. Both Tyndall/UU smart gloves have the potential to assist medical clinicians with precise measurements of the common condition loss of mobility in patients with RA [25].

O’Flynn et al. [48] results found that the IMU data glove had repeatable data similar to the UG, but with the removal of intra-tester and inter-tester reliability issues. Several studies by Connolly [22], O’Flynn et al. [42,48] and Fuchun Sun [52] found that IMUs are the key technologies to fiber optic devices used in the 5DT glove. Similarity, Connolly [22] found that the IMU glove had similar accuracy to the Vicon motion system, whilst requiring no calibration and having less overall error than the 5DT data glove. In addition, Wang et al. [56,64] findings indicate that IMUs can be used to monitor and measure ROM during rehabilitation. Moreover, they found that IMU-based gloves can record hand kinematics accurately and are lightweight and could be easily worn.

Data gloves are typically controlled with a microcontroller, which can translate many sensor outputs into a recognizable form of information to monitor hand function [44]. Commercialized data gloves are quite cost prohibitive compared to the traditional UG [23]. Depending on the sensor technology and the connection type between the glove and the computer collecting the data, different technologies can significantly increase the overall expense of the device [58]. Furthermore, a glove with wireless connectivity using a Bluetooth or WIFI module would benefit the wearer by allowing them to move around their home more freely [16]. Whilst commercially available data gloves are quite advanced and can provide a broad array of information, they may not be the best solution when only certain types of information are required in hand function [42]. Most of the aforementioned devices contain sensors mounted on cloth (textile) support known as the glove skin [42]. However, a common drawback in this approach is a lower level of comfort and an obtrusive nature of the glove as donning and doffing of the device is affected; it can cause obstructions to flexion/extension movements [45].

**Figure 27:**
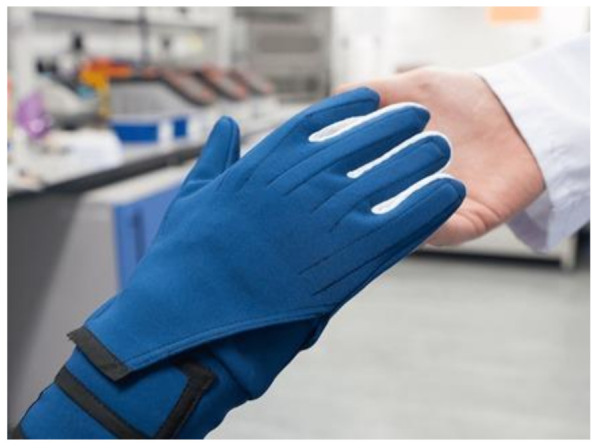
ActionSense smart glove [60].

More recent developments have led to the creation of a smart glove that examines the flexibility and limitation of an RA patient’s hand joints by measuring angular and velocity data [9]. The ActionSense team [60] have created the smart glove that contains multiple flex bend sensors that are placed in a textile substrate seen in Figure 27. Their clinical glove monitors the MCP and PIP joints, although the digits DIP joints, the thumbs CMC joint and the abduction and adduction movements between digits are not monitored. Hence, these movements are important to benefit the early recognition of RA [22,33,58,92].

**Figure 28:**
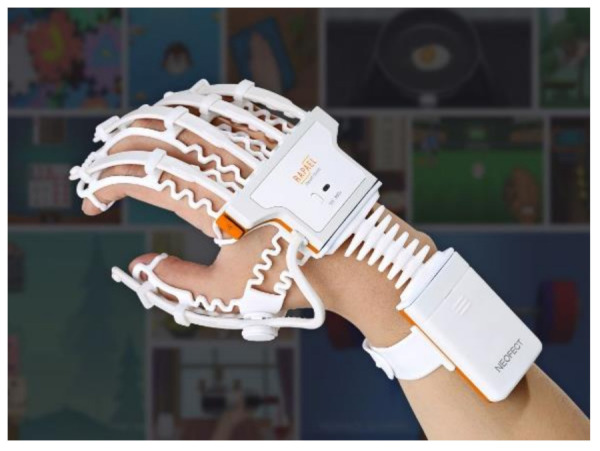
Neofect’s Rapael smart glove [93].

A high-tech exoskeleton device that fits over the patient’s hand and wrist, seen in Figure 28, was developed by Neofect to improve the patient’s neuroplasticity and to restore the function of stroke survivors [93,94]. The Rapael smart glove is a neurological rehabilitation system that is combined with a data-based digital activity system [16]. Furthermore, the smart device measures movement of the digits wrist and forearm using a combination of bend sensors and IMU’s [93,94]. Details of the level of accuracy, the finger joints measured, and the resolution of the device are limited or not available. However well the device works as a rehabilitative device for post stroke, its capabilities of assisting the diagnosis of RA patients may be sacrificed due to the sensor monitoring technique of the digits.

**Figure 29:**
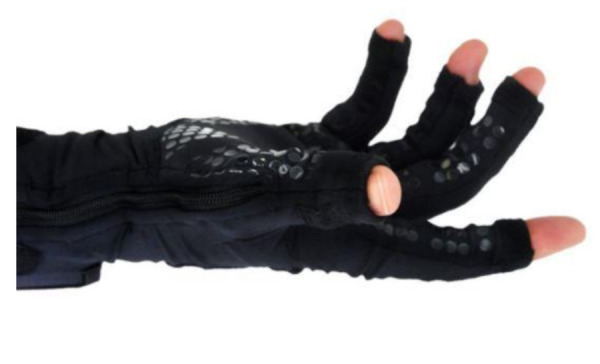
StretchSense [74].

A recent commercialized smart glove created by StretchSense (MoCap Pro SuperSplay) [74], seen in Figure 29, provides a solution for film and gaming animation [75]. The second generation of MoCap Pro gloves features a (variable capacitance) splay sensor with three sensing areas to capture the bend of each MCP and PIP joint as well as the sideways splay or finger spread of each digit. Additionally, the IP, MCP joints of the thumb and the abduction and adduction movements are monitored using the same 3-in-1 sensor all positioned in a textile substrate [74]. Generally, the outer segment of the sensor sits mainly over the PIP joint and across part of the DIP joint where the DIP value is ultimately an average of both.

**Figure 30:**
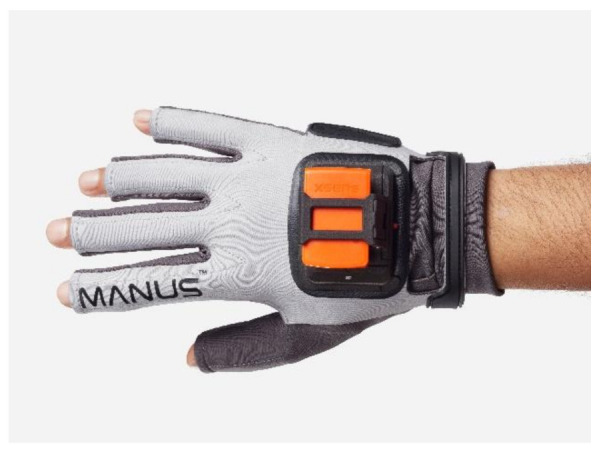
Manus Prime II Xsens) [95].

Finally, another recent commercialized glove (Manus Prime II Xsens) seen in Figure 30 provides finger tracking [95]. Their device supports 11 DOF for full finger spread tracking ensuring the finest motor movements. In addition, the Prime II Xsens gloves have been specially designed to work smoothly with Xsens software industry professionals around the world. Manus look to merge robustness with precision. Hence, all their Prime II series gloves contain industrial grade flex sensors placed across two joints of each finger [96]. Additionally, their Prime II Xsens device contains IMUs to ensure fine finger movement. IMU drift is avoided by their recently introduced automated filters, improved by the reference points of powerful flex sensors. This allows for detailed finger spread calculation without loss of continuous consistency during live performances. Their gloves are immune to magnetic interference.

**Figure 31:**
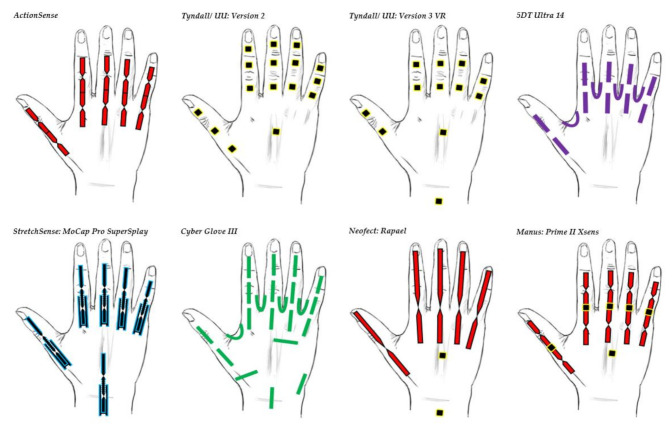
Modern commercialized and research smart gloves.

Table 2 and Figure 31 show a summary of recent commercialized and research smart gloves created by well-known research and commercial companies around the world. The commercial data gloves frequently use expensive motion analyzers and sensing fibers and can be quite costly for the consumer/health care market. Some research institutes (ActionSense) aim to lower the cost of the data glove for the health care industry, but this tends to have a negative impact on the overall accuracy and resolution of a device. In conclusion, several technologies have been proposed to monitor the hand over the years for HMI and in the clinical practices. They have been created with the expectation of generating accurate and reliable real time data measurements. Additionally, they provide more effective diagnosis procedures, whilst bringing efficient self-monitoring activities to a patient’s home. These technologies show potential for diagnosis and rehabilitation of neurological conditions, since gestures and movements are observed accordingly. Such hand functions can be detected based on both analog and digital technologies each with their own weakness and strengths.

### 3.1. Glove Materials

With any smart glove, a substrate material is used to contain all the sensors, wires, circuitry and other components [48]. One of the first questions that a designer or manufacturer of a wearable device needs to answer in the design process is: Can the product be worn on the hand [88,97]. As discussed in the earlier literature, the human hands come in all different shapes and sizes. Thus, this makes the design process quite challenging for the designers of wearable technology. Additionally, the substrate material must be designed to meet the demanding value in terms of wearability, hard wearing and washable [57]. In addition, the wearable material must include good skin adhesion, so that sensor accuracy is not disadvantaged, whilst being flexible and lightweight [44,58]. The Covid-19 pandemic has put an increased focus on the need for hand hygiene. To reduce any potential spread of the virus between glove users, the substrate material must be washable whilst having good chemical and physical stability during the washing process [58]. Moreover, the substrate material must allow the sensors, components and accessories to be removed beforehand if they do not meet the Ingress Protection (IP) IP68 requirements [98]. Water pretentious components must be removed if they are not protected whilst being immersed in water under pressure for a long period of time.

**Textile substrates**—Most of the sensory gloves reviewed in Table 2 are developed using a textile material such as, Lycra/spandex, nylon or other polymer materials which provides the desired mechanical properties for a smart glove [16,18,22,42,44,56]. However, in the modern era, nanotechnology can be used to create fabrics with unique functions for wearable technology, including UV defense, water and stain resistant, anti-bacterial, and much more [86,99]. This technology helps to incorporate the electrical elements into the textile. In addition, by manipulating the fabric surface, nanotechnology offers various efficient techniques and resources to produce the necessary fabric attributes. Moreover, demand for wearable devices and smart textiles in all segments has increased with the miniaturization of electronic components [100].

**Exoskeleton Design**—In recent commercialized products, the design developed by Neofect have moved in a different direction developing an exoskeleton rehabilitation glove [93,94]. Their glove is soft, flexible, allows the wrist to travel unrestrictedly, and leaves the palm free for grabbing and handling objects. Furthermore, an exoskeleton device has the components and sensors embedded onto the exoskeleton body, which closely follows the joints of the hand [101]. Lastly, such a device is clipped on to the hand and has the main advantages of being adjustable and easily wiped down without the need for any dismantling, unlike a textile glove [56].

### 3.2. Sensor and Device Calibration

Hand motion is an important component, which plays a key role in hand function assessment. As discussed in the literature, it is difficult to capture accurate hand motion, particularly in combination with sensor topologies placed on a textile or an exoskeleton device. Smart gloves require time-consuming and monotonous calibration to increase the devices overall accuracy during operation [5,29]. For this reason, frequent calibrations from data gloves require opening and closing the hand or even pre-set hand gesture parameters [102].

One constraint on the practical use of full-hand feedback is the challenge of understanding the motion. Hand tracking technology must take into consideration the context of the gesture and the movement of the participant’s hand position. The values recorded can differ depending on sensor resolution, hand size, sensor strain, human variations, and system interference [88]. Consequently, anthropometric dimensions have significant differences among individuals, including hand size, fingers length and thickness [33]. Glove-based systems must then be associated with various finger joint locations for various people, which would have an influence on the overall measurements [18,31]. Designers must have accurate data regarding the resolution, linearity, variability, and durability of the sensor information gathered by the sensory glove system before scalable and usable motion recognition applications can be written.

The accuracy and repeatability of data gloves is commonly compromised by the non-linear response of the sensors and any misalignment between the textile or non-textile holders between users. Calibration of the data glove sensor increases sensor accuracy and fits the limits of each sensor glove to those of the wearer. Moreover, a calibration routine involves the glove wearer to position their hand/finger joints in specific poses [42]. Typically, the wearer places their fingers at the minimum and maximum limits related to the data glove sensors. Calibration techniques imply that each finger joint can be shifted to its full finger joint position by the wearer. However, restricted joint mobility in RA sufferers render the data glove ineffective as they cannot attain the optimum movement [22].

The commercial glove 5DT requires calibration between every user in order to maintain the maximum sensitivity within an application. The range of each fiber optic sensor’s needs were set by the user flexing and unflexing each of their fingers. Furthermore, the calibration process is quite tedious as the user must keep the motion natural and unforced. Calibrations can be loaded at any time for a user but for optimum results, it is best to complete the calibration process every time the glove is put on [69]. Hence, as the 5DT sits snuggly to the user’s hand, and can be quite tricky to don and doff, as the positioning of the 14 sensors may not always be in the exact same position.

Recent innovations like the Stretchsense’s MoCap Pro SuperSplay gloves also require calibration [74]. However, much like 5DT, there is no need to constantly re-calibrate the gloves due to the stretchy snug textile surrounding the hand. The increased signal to noise ratio means that gloves can be calibrated even faster than the previous MoCap Pro because considerably fewer poses are necessary, saving time and money [75]. Additionally, there is a better probability of a good output and a good interaction between the data collected by the glove and the mocap software if a user does not train the device properly. Since the MoCap Pro SuperSplay sensors are non-optical, there are no issues in relation to occlusion, drift or constantly trying to re-calibrate your gloves like you would with an IMU device [42].

The ActionSense smart glove has five sensors, with each having two integrated flex sensors [9]. To calibrate each sensor, only two voltage points are required for this transducer typology which correspond to the flat hand position and the fist closed position [44]. As the sensors have only 1 DOF, this does benefit the calibration process unlike other sensors that output more detailed data. Other commercial data gloves, such as the CyberGlove III and Neofect’s Rapael glove, require similar monotonous sensor calibrations [88,103].

Data gloves that contain IMU typologies also require calibration in order to provide accurate orientation in three-dimensional space. Within these complex devices, IMUs contain triads of gyroscopes, accelerometers and magnetometers each requiring calibration [22,42,44,58]. Gyroscopes are prone to drift over a certain period of time and preventative corrections and calibrations are commonly used to combat this issue [5]. Some systems use various built-in drift-detection features such as sensor fusion [29]. For example, each sensor may be unreliable on its own, though when fused together with software, the overall system may become stable and accurate. Advanced smart gloves containing multiple IMUs may require a complete calibration process of all its devices, which can be quite tedious and substantially processing heavy [17].

Drawing attention to the work of Connolly et al. [42], their findings suggest that complex calibration is not needed for the IMU sensors on the iSEG-Glove. An IMU device mounted on each of the phalanges of the finger automatically provides information inside a complete sphere on the inclination and gravity orientation.

Depending on the sensor technology used within a device, a calibration method to set a distinctive sampling process of all the sensors may be required. A glove device that requires zero calibration would hugely benefit its use in and out of clinical practice for RA assessment.

## 4. Discussion

Recognizing early signs of RA from similar diseases at the onset of illness is not easy, as issues related to hand function assessment exist. Clinicians and researchers and have found issues related to inter-rater and intra-rater reliability measurement when using a hand-held UG. Research on the effectiveness of the UG report a difference of 7–9° between therapists when measuring joint ROM, leading to a 27° difference over the three finger joints. More detailed and less laborious approaches are required to record joint movements if patients are to get proper treatment for their condition.

This study reviews various data gloves developed since the early 1970’s, focusing on the key features of glove-based systems for hand function assessment. In the modern era, advanced technologies have been developed and implemented to resolve issues related to UG, but are constrained in terms of costs, wearability and accuracy. Various electronic sensors and devices have been proposed in these studies, which include: flex bend, fiber optic, IMU/accelerometer, gyroscope, magnetometer, capacitive and Hall effect sensors. IMUs tend to be the most extensively used sensing devices as they yield reliable critical values precisely, whilst providing useful data like acceleration, angular velocity, magnetic field and ROM as manual dexterity parameters for the physicians.

Many researchers have only conducted static tests on data gloves and sensors, making the glove validation process restricted. Several researchers have used non-contact approaches to measure contact systems. This hinders the end results as non-contact systems [49] suffer from inaccuracy due to self-occlusion, environmental conditions and human error at the set up. Other researchers have used wooden blocks [22] and custom basic sensor testing systems [29,49] that already have a high rate of error within them. Without a high precision and a dynamic authentication system, sensor verification for a hand-monitoring device is unsatisfactory as movements do not mimic that of the hand function ability or finger joint movement.

Some new nanotechnology on innovative sensing technologies provide new solutions for a wide range of health assessments, providing new physical sensing solutions that allow for improved sensor performance, accuracy and flexibility. However, they have been excluded from the survey as they are only sensing technologies that have not been applied to past or current data gloves. Nanotechnology addresses the potential to develop a new generation of sensors and the difficulties of wearable electronics to keep up with the increasingly prominent need for nanostructured materials.

Throughout the research, there is lack of evidence showing truthful performance of all sensors/technologies used to assess hand function. Therefore, a benchmark system is needed that closely mimics hand joint movement to evaluate all technologies dynamically and systematically.

## 5. Conclusions

The improvement in hand function is reported and completing general exercises is evidently reducing pain, stiffness and swelling of the hand. Sensory gloves have been used in several applications, such as robotics and VR, but researchers have turned their attention towards medical applications in recent years. This research emphasizes the importance of early RA diagnoses and rehabilitation methods. It is necessary to recognize RA at its early stages in order to start treatment. Existing methods of detecting RA rely on observation, questionnaires, and physical measurement, each having their own weaknesses. A lot of research has been conducted by multiple organizations over a number of years; researchers and clinicians have worked together to standardize the classifiers of RA and to try to make the clarification of results identical; however, this is currently not the case. Clinicians and researchers have established ROM as the main measure of joint stiffness. However, there still is a great deal of ambiguity between patient diagnosis as the difficultly of measurement increases throughout patients.

Morning stiffness is currently not measured by Rheumatologists as it is only feasible to measure in the first 30 min of a person’s day. Sensor gloves have the capability of remotely monitoring morning stiffness and the assessment of hand function, although their realistic measures are still limited by accuracy, weight, size, wearability, and cost. This investigation continues and focuses on the main issues to overcome the limitations of current research and commercially available gloves.

Further in-depth research needs to be conducted into various types of sensors currently available for data gloves; resistance transducers, fiber optic bend sensors, IMUs, capacitive bend sensors and Hall effect sensors. It is important that the sensors are tested for static and dynamic movement away from the glove skin first to determine the sensors physical characteristics. Once this is complete, further investigation may be needed while placing the sensors on the glove skin. This research continues investigation of contact systems, carefully considering various technologies and how they can assist with the diagnosis of RA conditions.

In conclusion, research needs to be carried out in the area of user personalization through design and production to ensure characteristics of the medical glove applications are more efficient and accurate. To clarify, when accurate sensor characteristics are monitored and validated, the selection of the sensors for a novel smart glove to assist with RA and hand functional assessment can be finalized.

## 6. Future Work

A benchmark system that closely mimics hand joints dynamic movement will be produced. Please see Supplementary Material (ECSMS demo, Video S1). It is important that the system is validated to a high level of accuracy to test advanced sensor technologies and to retrieve their real characteristics. The system will perform non-obstructive rotary movements on sensors alone, sensors on/in different materials and sensors on a dummy finger with a glove device attached. Once the results are gathered using such a system [104], several statistical tests will be performed on the sensors repeatability and accuracy tests to determine how reliable they are.

## Figures and Tables

**Table 1:** Comparison of Popular Linear Sensors.

Figure	Device/Sensor	Cost Device	Individual Sensor	DOF	Voltage Supply	Output Type
**Figure 5.**	Flexpoint 2 in 1 sensor	N/A	£5–£20	1 DOF	3.3 V–12 V	Analog
**Figure 10.**	Fibre optic sensor	N/A	-	1 DOF	IR = 1.2 V Photodiode = 2.5 V	Analog
**Figure 11.**	Hall effect sensor	N/A	£1–£5	1 DOF	4.5 V–10.5 V	Analog, Ratiometric
**Figure 13.**	StretchSense capacitive sensor	N/A	-	3 DOF	0–3 V	Analog
**Figure 18.**	Adafruit MPU-6050	£10	£7	6 DOF Acc & Gyro	2.375 V–3.46 V	Digital, I2C (400 kHz) 16-bit
**Figure 19.**	SparkFun LSM9DS1	£10	£2.78	9 DOF Acc & Gyro & Mag	1.9 V–3.6 V	Digital, I2C (400 kHz)/SPI 16-bit
**Figure 20.**	Adafruit MPU-9250	£7	£5–£8	9 DOF Acc & Gyro & Mag	2.4 V–3.6 V	Digital, I2C (400 kHz)/ SPI 16-bit

**Table 2:** Modern commercialized and research smart gloves.

Data Glove	Use	Market	Number of Sensors	Cost	Sensor Technology	Sensor Description	Joints Monitored(Refer to All Glove Image for Illustration)	Legend
**5DT Ultra 14**	Motion capture and animation	Commercial	14	£5000	Fibre Optic	5DT’s own sensor	All MCP & PIP joints of fingers. MCP & IP joints of thumb. Splay of all digits.	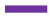
**Tyndall/UU: Version 2**	Clinical	Research	16	£26,000	IMU (9 DOF)	MPU-9150	All MCP, PIP & DIP joints of fingers. IP, MCP & CMC joints of thumb. Splay of all digits.	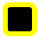
**Tyndall/UU: Version 3 VR**	Clinical/VR	Research	12	£12,000	IMU (9 DOF)	MPU-9250	All MCP & PIP joints of fingers. IP & MCP joints of thumb. Splay of all digits & wrist movement.	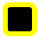
**Cyber Glove III**	Motion capture environment	Commercial	22	$15,000	Flex bend	Unknown	All MCP, PIP & DIP joints of fingers. IP, MCP & CMC joints of thumb. Splay of all digits, wrist & palm arch movement.	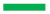
**ActionSense**	Clinical	Research	5	£400	Flex bend	Flexpoint’s 2in1 combined sensor	All MCP & PIP joints of fingers. IP & MCP joints of thumb.	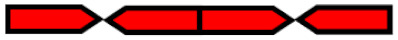
**Neofect: Rapael**	Clinical	Commercial	5 Flex bend 2 IMU	$15,000	Flex bend & IMU	Unknown	Fingers, wrist & forearm movement.	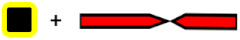
**Manus: Prime II Xsens**	Character animation	Commercial	10 flex bend 5 IMU	$5000–$6000	Flex bend & IMU (Combined sensor fusion)	Unknown	All MCP & PIP joints of fingers. IP, MCP & CMC joints of thumb.	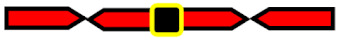
**StretchSesne: MoCap Pro SuperSplay**	Film and game animation	Commercial	6	$7150	Capacitive	StretchSense’s own SuperSplay sensor	All MCP & PIP joints of fingers. MCP & IP joints of thumb. Splay of all digits & wrist movement.	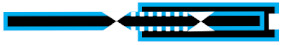

**Note:** Please refer to Figure 31 for sensor positioning and arrangement on the hand joints.

## Data Availability

Not applicable

## Supplementary Materials

The following are available online at https://youtu.be/yFF0IhfUQFc (Video S1).

## References

[1] Rashid A., Hasan O. (2019). Wearable technologies for hand joints monitoring for rehabilitation: A survey. Microelectron. J..

[2] Institute for Quality and Efficiency in Health Care (2010). How do hands work?. NCBI.

[3] Salawu F.K., Danburam A., Olokoba A.B. (2010). Non-motor symptoms of Parkinson’s disease: Diagnosis and management. Niger. J. Med..

[4] Baldominos A., Saez Y., del Pozo C.G. (2015). An Approach to Physical Rehabilitation Using State-of-the-art Virtual Reality and Motion Tracking Technologies. Procedia Comput. Sci..

[5] Davarzani S., Pajouh M.A.A. Design and Fabrication of Sensing System for Rehabilitation of Finger. Proceedings of the 2020 28th Iranian Conference on Electrical Engineering (ICEE).

[6] Brigante C.M.N., Abbate N., Basile A., Faulisi A.C., Sessa S. (2011). Towards miniaturization of a MEMS-based wearable motion capture system. IEEE Trans. Ind. Electron..

[7] Orbai A.M., Smith K.C., Bartlett S.J., de Leon E., Bingham C.O. (2014). ‘Stiffness Has Different Meanings, I Think, to Everyone’: Examining Stiffness from the Perspective of People Living With Rheumatoid Arthritis. Arthritis Care Res..

[8] Rajak R., Zaman M., Jones T., Sheikh F., Sharif M. (2019). Thu0617 Wrist Ultrasound (Us) Pathology in Early Rheumatoid Arthritis (Ra); Observations from an Early Inflammatory Arthritis (Eia) Diagnostic Service. Ann. Rheum. Dis..

[9] Abbas S., Condell J., Gardiner P., McCann M., Todd S., Connolly J. Can multiple wearable sensors be used to detect the early onset of Parkinson’s Disease?. In Proceedings of the 2020 31st Irish Signals and Systems Conference (ISSC).

[10] Majithia V., Geraci S.A. (2007). Rheumatoid Arthritis: Diagnosis and Management. Am. J. Med..

[11] Johnson J. (2019). Symmetric vs. asymmetric arthritis: What to know. Medical News Today.

[12] Bukhari M., Kent A. (2020). How rheumatologists assess disability in the current era needs an overhaul: Focus on the Health Assessment Questionnaire. Rheumatology (Oxford).

[13] Hall T.C., Nixon M.F., Dias J.J., Graham T., Cook S. (2010). How accurately does a simulation glove reflect function compared to rheumatoid arthritis sufferers?. Ann. R. Coll. Surg. Engl..

[14] Rat A.C., Boissier M.C. (2004). Rheumatoid arthritis: Direct and indirect costs. Jt. Bone Spine.

[15] Smolen J.S., Landewé R., Bijlsma J., Burmester G., Chatzidionysiou K., Dougados M., Nam J., Ramiro S., Voshaar M., Van Vollenhoven R. (2017). EULAR recommendations for the management of rheumatoid arthritis with synthetic and biological disease-modifying antirheumatic drugs: 2016 update. Ann. Rheum. Dis..

[16] Raad M.W., Deriche M.A., Hafeedh A.B., Almasawa H., Jofan K.B., Alsakkaf H., Bahumran A., Salem M. (2019). An IOT based wearable smart glove for remote monitoring of rheumatoid arthritis patients. BIOSIGNALS.

[17] Debes C., Merentitis A., Sukhanov S., Niessen M., Frangiadakis N., Bauer A. (2016). Monitoring activities of daily living in smart homes: Understanding human behavior. IEEE Signal Process. Mag..

[18] Nasir S.H., Troynikov O., Westropp N.M. (2014). Therapy gloves for patients with rheumatoid arthritis: A review. Ther. Adv. Musculoskelet. Dis..

[19] Mäkinen H., Kautiainen H., Hannonen P., Sokka T. (2005). Is DAS28 an appropriate tool to assess remission in rheumatoid arthritis?. Ann. Rheum. Dis..

[20] NRAS (2017). The DAS28 score. National Rheumatoid Arthritis Society.

[21] Van Riel P.L. (2014). The development of the disease activity score (DAS) and the disease activity score using 28 joint counts (DAS28). Clin. Exp. Rheumatol..

[22] Connolly J. (2015). Wearable Rehabilitative Technology for the Movement Measurement of Patients with Arthritis.

[23] Milanese S., Gordon S., Buettner P., Flavell C., Ruston S., Coe D., O’Sullivan W., McCormack S. (2014). Reliability and concurrent validity of knee angle measurement: Smart phone app versus universal goniometer used by experienced and novice clinicians. Man. Ther..

[24] North Coast Medical (2020). Hand Goniometer. North Coast Medical.

[25] Ghosh S. (2013). Capturing Human Hand Kinematics for Object Grasping and Manipulation.

[26] Lorig K.R., Mazonson P.D., Holman H.R. (1993). Evidence suggesting that health education for self-management in patients with chronic arthritis has sustained health benefits while reducing health care costs. Arthritis Rheum..

[27] Cheung P.P., Gossec L., Mak A., March L. (2014). Fc literature review. Semin. Arthritis Rheum..

[28] Keogh J.W., Cox A., Anderson S., Liew B., Olsen A., Schram B., Furness J. (2019). Reliability and validity of clinically accessible smartphone applications to measure joint range of motion: A systematic review. PLoS ONE.

[29] Lin B.S., Lee I.J., Yang S.Y., Lo Y.C., Lee J., Chen J.L. (2018). Design of an Inertial-Sensor-Based Data Glove for Hand Function Evaluation. Sensors.

[30] Salter N. (1955). Methods of measurement of muscle and joint function. J. Bone Joint Surg. Br..

[31] Burr N. (2003). Inter-rater and Intra-rater Reliability when Measuring Interphalangeal Joints. Physiotherapy.

[32] Ellis B., Bruton A. (2002). A study to compare the reliability of composite finger flexion with goniometry for measurement of range of motion in the hand. Clin. Rehabil..

[33] Lee K.S., Jung M.C. (2015). Ergonomic evaluation of biomechanical hand function. Saf. Health Work.

[34] Kerschan-Schindl K., MacHold K. (2011). Rehabilitation von Patienten mit rheumatoider Arthritis. Phys. Medizin Rehabil. Kurortmed..

[35] Carteron N. (2020). What are the symptoms of rheumatoid arthritis?. Healthline.

[36] (2020). Arthritus Foundation, How Rheumatoid Arthritis Affects More Than Joints. https://www.arthritis.org/diseases/more-about/how-rheumatoid-arthritis-affects-more-than-joints.

[37] Bakir E., Samancioglu S., Gursoy S. (2018). Complementary Therapies in Clinical Practice the effects of re fl exology on pain and sleep deprivation in patients with rheumatoid arthritis: A randomized controlled trial. Complement. Ther. Clin. Pract..

[38] Metsios G.S., Kitas G.D. (2019). Best Practice & Research Clinical Rheumatology Physical activity, exercise and rheumatoid arthritis: Effectiveness, mechanisms and implementation. Best Pract. Res. Clin. Rheumatol..

[39] Rapoliene J., Krisciunas A. (2006). The effectiveness of occupational therapy in restoring the functional state of hands in rheumatoid arthritis patients. Medicina (Kaunas).

[40] Dipietro L., Sabatini A.M., Dario P. (2003). Evaluation of an instrumented glove for hand-movement acquisition. J. Rehabil. Res. Dev..

[41] O’Flynn B., Torres J., Connolly J., Condell J., Curran K., Gardiner P. Novel smart sensor glove for arthritis rehabiliation. Proceedings of the 2013 IEEE International Conference on Body Sensor Networks.

[42] Connolly J., Condell J., O’Flynn B., Sanchez J.T., Gardiner P. (2018). IMU Sensor-Based Electronic Goniometric Glove for Clinical Finger Movement Analysis. IEEE Sens. J..

[43] Lin B.S., Lee I.J., Chen J.L. (2020). Novel Assembled Sensorized Glove Platform for Comprehensive Hand Function Assessment by Using Inertial Sensors and Force Sensing Resistors. IEEE Sens. J..

[44] de Pasquale G. (2018). Glove-based systems for medical applications: Review of recent advancements. J. Text. Eng. Fash. Technol..

[45] Kumar S., Sultan M.J., Ullah A., Zameer S., Siddiqui S., Sami S.K. (2018). Human Machine Interface Glove Using Piezoresistive Textile Based Sensors. IOP Conf. Ser. Mater. Sci. Eng..

[46] Mori Y., Toyonaga M. Data-glove for japanese sign language training system with gyro-Sensor. Proceedings of the 2018 Joint 10th International Conference on Soft Computing and Intelligent Systems (SCIS) and 19th International Symposium on Advanced Intelligent Systems (ISIS).

[47] Pham T., Pathirana P.N., Trinh H., Fay P. (2015). A non-contact measurement system for the range of motion of the hand. Sensors.

[48] O’Flynn B., Sachez-Torres J., Tedesco S., Downes B., Connolly J., Condell J., Curran K. (2015). Novel Smart Glove Technology as a Biomechanical Monitoring Tool. Sens. Transducers.

[49] Lin B.S., Lee I.J., Chiang P.Y., Huang S.Y., Peng C.W. (2019). A Modular Data Glove System for Finger and Hand Motion Capture Based on Inertial Sensors. J. Med. Biol. Eng..

[50] Ruffing V. (2020). Rheumatoid Arthritis Signs and Symptoms. Johns Hopkins Arthritis Center.

[51] Netto A.P. (2015). Hand Pain and Rheumatoid Arthritis (RA). Veritas Health.

[52] Fang B., Sun F., Liu H., Guo D. (2015). A Novel Data Glove Design Based on Inertial and Magnetic Sensors. Int. J. Swarm Intell. Evol. Comput..

[53] Ding S., Schumacher M. (2016). Sensor monitoring of physical activity to improve glucose management in diabetic patients: A review. Sensors.

[54] Das A., Yadav L., Singhal M., Sachan R., Goyal H., Taparia K., Gulati R., Singh A., Trivedi G. Smart glove for sign language communications. Proceedings of the 2016 International Conference on Accessibility to Digital World (ICADW).

[55] Braun A., Wichert R., Kuijper A., Fellner D.W. (2014). A benchmarking model for sensors in smart environments. European Conference on Ambient Intelligence.

[56] Wang Q., Markopoulos P., Yu B., Chen W., Timmermans A. (2017). Interactive wearable systems for upper body rehabilitation: A systematic review. J. Neuroeng. Rehabil..

[57] Routhier F., Duclos N.C., Lacroix É., Lettre J., Turcotte E., Hamel N., Michaud F., Duclos C., Archambault P.S., Bouyer L.J. (2020). Clinicians’ perspectives on inertial measurement units in clinical practice. PLoS ONE.

[58] Faisal A.I., Majumder S., Mondal T., Cowan D., Naseh S., Deen M.J. (2019). Monitoring methods of human body joints: State-of-the-art and research challenges. Sensors.

[59] Ahmed M.A., Zaidan B.B., Zaidan A.A., Salih M.M., Lakulu M.M.B. (2018). A review on systems-based sensory gloves for sign language recognition state of the art between 2007 and 2017. Sensors.

[60] Condell J., Connolly J., Young W. (2020). Action Sense. https://www.actionsense.org/.

[61] (2020). Flex Point. https://www.flexpoint.com/bend-sensor.

[62] Saggio G., Lagati A., Orengo G. (2012). Shaping Resistive Bend Sensors to Enhance Readout Linearity. ISRN Electron..

[63] Components 101 Flexpoint characteristics. Components 101.

[64] Wang Q., Liu Y. (2018). Review of optical fiber bending/curvature sensor. Meas. J. Int. Meas. Confed..

[65] Remouche M., Georges F., Meyrueis P. (2013). Stress Sensing by an Optical Fiber Sensor: Method and Process for the Characterization of the Sensor Response Depending on Several Designs. Opt. Photonics J..

[66] Ivanov O.V., Chertoriyskiy A.A. (2015). Fiber-Optic Bend Sensor Based on Double Cladding Fiber. J. Sens..

[67] Tyndall National Institute (2017). VR Glove. Tyndall National Institute.

[68] Tyndall National Institute (2015). Tyndall IMU version 2. Tyndall National Institute.

[69] 5th Dimention Technoligies (2020). 5DT Hand Book. 5DT.

[70] Ceruti M., Duffy L., Phan H., Eppele K. (2013). Hall Effect Glove. US Patent.

[71] Honeywell (2020). Hall efffect sensor SS495B. Honeywell.

[72] Lee Y.Y., Wu R.H., Xu S.T. Applications of linear Hall-effect sensors on angular measurement. Proceedings of the 2011 IEEE International Conference on Control Applications (CCA).

[73] Abraham L., Urru A., Normani N., Wilk M.P., Walsh M., O’flynn B. (2018). Hand tracking and gesture recognition using lensless smart sensors. Sensors.

[74] StretchSense (2020). StretchSense-MoCap Pro. StrectchSense.

[75] TEGARA (2020). StretchSense MoCap Pro SuperSplay Gloves, a glove for hand motion capture equipped with a high-precision sensor that detects expansion and contraction. https://www.tegakari.net/en/2020/08/stretchsense-mocap-pro-supersplay/.

[76] Shin Jeong Park (2020). StretchSense Characteristics. https://softroboticstoolkit.com/stretchsense.

[77] Neely J.S., Restle P.J. (1997). Capacitive Bend Sensor. US Patent.

[78] Analog Devices (2020). ADXL345. https://www.sparkfun.com/datasheets/Sensors/Accelerometer/ADXL345.pdf.

[79] Analog Devices (2020). ADXL335. https://www.analog.com/media/en/technical-documentation/data-sheets/adxl335.pdf.

[80] TDX Invensense (2020). MPU-6050. https://invensense.tdk.com/wp-content/uploads/2015/02/MPU-6000-Datasheet1.pdf.

[81] TDX Invensense (2020). Motion tracking MPU-6050. https://invensense.tdk.com/products/motion-tracking/6-axis/mpu-6050/.

[82] ST Electronics (2020). LSM9DS1. https://www.st.com/resource/en/datasheet/lsm9ds1.pdf.

[83] TDX Invensense (2020). MPU-9250. https://invensense.tdk.com/wp-content/uploads/2015/02/PS-MPU-9250A-01-v1.1.pdf.

[84] Sbernini L., Quitadamo L.R., Riillo F., di Lorenzo N., Gaspari A.L., Saggio G. (2018). Sensory-Glove-Based Open Surgery Skill Evaluation. IEEE Trans. Human-Machine Syst..

[85] Farnell (2013). Arduino Uno Datasheet. https://www.farnell.com/datasheets/1682209.pdf.

[86] Wu H., Guo H., Su Z., Shi M., Chen X., Cheng X., Han M., Zhang H. (2018). Fabric-based self-powered noncontact smart gloves for gesture recognition. R. Soc. Chem..

[87] Sturman D.J., Zeltzer D. (1994). A Survey of Glove-based Input. IEEE Sens. J..

[88] Kessler G.D., Hodges L.F., Walker N. (1995). Evaluation of the CyberGlove as a Whole-Hand Input Device. ACM Trans. Comput. Interact..

[89] Cyber Glove System (2020). Cyberglove III. http://www.cyberglovesystems.com/cyberglove-iii/.

[90] (2019). Pascal Lee, An Astronaut Smart Glove to Explore the Moon, Mars and Beyond. https://www.seti.org/press-release/astronaut-smart-glove-explore-moon-mars-and-beyond.

[91] (2020). 5th Dimention Technoligies, 5DT. https://5dt.com/5dt-data-glove-ultra/.

[92] Haroon A., Fergus P., Shaheed A., Merabti M. A wireless home and body sensor network platform for the early detection of arthritis. Proceedings of the 2010 7th IEEE Consumer Communications and Networking Conference.

[93] Neofect (2020). Rapael smart glove. https://www.neofect.com/us/blog/stroke-rehabilitation-is-now-fun-thanks-to-rapael-smart-glove.

[94] Rico P. (2020). Meditech, LLC. https://irp-cdn.multiscreensite.com/68072aa0/files/uploaded/RAPAELCatalogue_Eng.pdf.

[95] Manus (2020). Manus Prime 2 Xsens. https://manus-vr.com/xsens-gloves.

[96] Manus (2020). Manus Prime II Xsens. https://www.tegakari.net/en/2020/06/manus_prime_2/.

[97] Carbonaro N., Mura G.D., Lorussi F., Paradiso R., de Rossi D., Tognetti A. (2014). Exploiting wearable goniometer technology for motion sensing gloves. IEEE J. Biomed. Heal. Inform..

[98] Griffith E. (2020). Ingress Protection (IP) IP68 requirements. https://www.pcmag.com/how-to/dust-resistant-waterproof-making-sense-of-gadget-ratings.

[99] Initiative N.S. (2012). Nanotechnology for Sensors and Sensors for Nanotechnology: Improving and Protecting Health, Safety, and the Environment. Nanotechnol. Signat. Initiat..

[100] Goncu-Berk G., Topcuoglu N. (2017). A Healthcare Wearable for Chronic Pain Management. Design of a Smart Glove for Rheumatoid Arthritis. Des. J..

[101] Stilli A., Cremoni A., Bianchi M., Ridolfi A., Gerii F., Vannetti F., Wurdemann H.A., Allotta B., Althoefer K. AirExGlove-A novel pneumatic exoskeleton glove for adaptive hand rehabilitation in post-stroke patients. Proceedings of the 2018 IEEE International Conference on Soft Robotics (RoboSoft).

[102] Fujiwara E., Miyatake D.Y., Santos M.F.M.D., Suzuki C.K. Development of a glove-based optical fiber sensor for applications in human-robot interaction. Proceedings of the 2013 8th ACM/IEEE International Conference on Human-Robot Interaction (HRI).

[103] Innovation Channels (2020). Smart Glove to Make Stroke Rehab More Effective and Affordable. Innovation Enterprise Channel.

[104] Henderson J., Condell J., Connolly J., Kelly D., Curran K. (2021). Reliability and Validity of Clinically Accessible Smart Glove Technologies to Measure Joint Range of Motion. Sensors.

